# Supplementary material for: Online searches to evaluate misinformation can increase its perceived veracity
Source: Nature. 2023 Dec 20;625(7995):548–56. doi: 10.1038/s41586-023-06883-y (PMC10794132; doi:10.1038/s41586-023-06883-y)
Supplement: Supplementary file 1 — Supplementary Information [file 41586_2023_6883_MOESM1_ESM.pdf]

nature portfolio

https://doi.org/10.1038/s41586-023-06883-y

Supplementary information

Online searches to evaluate misinformation can increase its perceived veracity

In the format provided by the authors and unedited

Supplementary Information: Online Searches to Evaluate Misinformation Can Increase Its Perceived Veracity

Contents

A Articles Selected for Each Study **2**
A.1 Study 1 . . . 2
A.2 Studies 2 and 3 . . . 6
A.3 Study 4 . . . 13
A.4 Study 5 . . . 16

B Model Results for Figures 1–4 in Main Text **20**

C Descriptive Statistics for NewsGuard Ratings **64**

D Explanation of Sampling Technique for Respondents **65**

E Article Selection Process **66**
E.1 Mainstream News Sites We Sourced From . . . 66
E.2 Low-Quality News Sites We Sourced From . . . 67
E.3 Method for Determining Partisan Lean of News Sites . . . 72

F Attention Checks **73**

G Social Media Presence of Articles Selected in Studies 1–3 **74**

H Additional Instructions to Search Online for Information **75**

I Data Measurement – Studies 1–4 **76**

J Different Data Measurement – Study 5 **78**

K Fact-Checker Agreement **79**

L Ideological Perspectives of Articles (Studies 1–4) **80**

M Figures Only Using Robust Modal Classification **81**

N Number of Unique Respondents and Evaluations in Studies 1-5 **85**

O Study Using Different Instructions (Study 6) **87**

P Search Terms and Quality of News Sources Returned **96**

Q Balance Tables **101**

A Articles Selected for Each Study

A.1 Study 1

Table S1: Headlines for Articles Chosen from the Low Quality Liberal News Stream in Study 1

	Date	Headline	Modal Fact Checker Rating	Topic	Lean of Article
1	11/20/19	Lt. Col. Vindman: ‘This Is America...Here, Right Matters’	True	Political/Economy	Liberal
2	11/21/19	Sondland’s testimony directly implicates Trump, Pence and Pompeo in Ukraine quid pro quo plot	True	Political/Economy	Liberal
3	12/3/19	The sealed “Indictment A” that Donald Trump needs to worry about more than ever	False/Misleading	Political/Economy	Liberal
4	12/4/19	Devin Nunes Shamelessly Lies When Hannity Asks About Lev Parnas	False/Misleading	Political/Economy	Liberal
5	12/5/19	Trump caught by reporters patting himself on back for insulting Justin Trudeau	True	Political/Economy	Liberal
6	12/9/19	Ex-Intel Slam Trump For Sucking Up To Saudis After Navy Shooting	True	Political/Economy	Liberal
7	12/10/19	Nancy Pelosi knows something we don’t	False/Misleading	Political/Economy	Neutral
8	12/11/19	Tucker Carlson’s White Power Hour Guest: AOC’s District Is The ’Least American’	True	Political/Economy	Liberal
9	1/6/20	Schiff Hammers President & GOP Over Impeachment Trial Obstruction	True	Political/Economy	Liberal
10	1/7/20	Everything is falling apart for Donald Trump in real time	True	Political/Economy	Liberal

Table S2: Headlines for Articles Chosen from the Low Quality Conservative News Stream in Study 1

	Date	Headline	Modal Fact Checker Rating	Topic	Lean of Article
1	11/20/19	No Shots Fired! Citizen with a Gun Ends Gunman's Attack at Oklahoma Walmart	False/Misleading	Political/Economy	Conservative
2	11/21/19	Indictment Against Head Of Burisma Reveals 'Hunter Biden Was Receiving Payments From Money Raised Through Criminal Means, Siphoned, Laundered From Ukraine'	False/Misleading	Political/Economy	Conservative
3	12/3/19	Donald Trump SLAMS Corbyn's NHS lies 'We want nothing to do with it!'	False/Misleading	Political/Economy	Neutral
4	12/4/19	In 2018, 86% of Those Arrested for Violent Crime in Los Angeles Were Non-White (5% Were White): the City Is 28% White	False/Misleading	Political/Economy	Conservative
5	12/5/19	DING! DING! DING! First Muslim woman elected to Pennsylvania House of Representatives has been ARRESTED for stealing $500,000 from a charity	True	Political/Economy	Conservative
6	12/9/19	NEVER TRUMPER RICK WILSON SUGGESTS PUTTING ANTI-VAXXERS IN "RE-EDUCATION CAMPS"	True	Political/Economy	Conservative
7	12/10/19	Breaking: Ukrainian Official Reveals Six Criminal Cases Opened In Ukraine Involving The Bidens	False/Misleading	Political/Economy	Conservative
8	12/11/19	Ukraine Advisor Disputes Key Point In Impeachment Testimony — Is This Bad News For Democrats?	False/Misleading	Political/Economy	Conservative
9	1/6/20	NEARLY 200 PEOPLE ARRESTED ACROSS AUSTRALIA FOR DELIBERATELY STARTING BUSHFIRES	False/Misleading	Science	Conservative
10	1/7/20	Iran stampede: '35 dead' and dozens injured after huge crush at Qassem Soleimani funeral	True	Political/Economy	Neutral

Table S3: Headlines for Articles Chosen from the Mainstream Conservative News Stream in Study 1

	Date	Headline	Modal Fact Checker Rating	Topic	Lean of Article
1	11/20/19	Key impeachment witness dodges GOP questions to protect whistleblower	True	Political/Economy	Neutral
2	11/21/19	Smollet Claims He Suffered 'Extreme Emotional Distress' in Malicious Prosecution Lawsuit Against Chicago	True	Human Interest	Neutral
3	12/3/19	Marine veteran turned congressional candidate calls Kaepernick a 'national disgrace'	True	Political/Economy	Conservative
4	12/4/19	Devin Nunes slaps CNN with $435 million defamation lawsuit	True	Political/Economy	Neutral
5	12/5/19	Angry Melania Slams Impeachment Witness for Joking About Son	True	Political/Economy	Conservative
6	12/9/19	Walmart apologizes for sweater featuring Santa with cocaine	True	Human Interest	Neutral
7	12/10/19	Joe Biden Claims No One Told Him About Potential Conflict of Interest With Hunter's Job at Burisma	True	Political/Economy	Conservative
8	12/11/19	House Democrats announce articles of impeachment against Trump: Abuse of power, obstruction of Congress	True	Political/Economy	Neutral
9	1/6/20	Ricky Gervais blasts Hollywood figures as unprincipled, ignorant at Golden Globes	True	Human Interest	Neutral
10	1/7/20	Pelosi Says the House Will Vote on a Resolution to Limit Trump's Military Actions Regarding Iran	True	Political/Economy	Neutral

Table S4: Headlines for Articles Chosen from the Mainstream Liberal News Stream in Study 1

	Date	Headline	Modal Fact Checker Rating	Topic	Lean of Article
1	11/20/19	Woman Saves Scorched Koala From Bushfire With Shirt Off Her Own Back	True	Science	Neutral
2	11/21/19	Almaas Elman, Somali-Canadian Activist, Is Shot Dead in Mogadishu	True	Political/Economy	Neutral
3	12/3/19	Duncan Hunter To Plead Guilty In Campaign Finance Case He Called 'Witch Hunt'	True	Political/Economy	Neutral
4	12/4/19	Kamala Harris Dropping Out Of Presidential Race	True	Political/Economy	Neutral
5	12/5/19	'He Showed Us Life': Japanese Doctor Who Brought Water to Afghans Is Killed	True	Human Interest	Neutral
6	12/9/19	Caroll Spinney, legendary 'Sesame Street' puppeteer of Big Bird, dies at 85	True	Human Interest	Neutral
7	12/10/19	Megan Rapinoe is Sports Illustrated's Sportsperson of the Year, only the fourth woman chosen alone	True	Human Interest	Neutral
8	12/11/19	Police Chief Tears Into Ted Cruz, McConnell For Caring More About NRA Than Gun Victims	True	Political/Economy	Neutral
9	1/6/20	Mike Pence Slammed After Falsely Linking Qassem Soleimani To 9/11	True	Political/Economy	Liberal
10	1/7/20	Pentagon Rules Out Striking Iranian Cultural Sites, Contradicting Trump	True	Political/Economy	Liberal

Table S5: Headlines for Articles Chosen from the Low Quality Unclear News Stream in Study 1

	Date	Headline	Modal Fact Checker Rating	Topic	Lean of Article
1	11/20/19	Pounds lost doesn't mean FAT lost: You CAN lose up to 2 pounds of fat a month – but it takes consistency and patience	False/Misleading	Science	Neutral
2	11/21/19	Ukrainian MP Claims $7.4 Billion Obama-Linked Laundering, Puts Biden Group Take At $16.5 Million	False/Misleading	Political/Economy	Conservative
3	12/3/19	Americans Bought Enough Guns on Black Friday to Arm the Marine Corps – Yet Again!	True	Political/Economy	Unclear
4	12/4/19	Ukrainian Neo-Nazis Help Out at Hong Kong Riots, Pan-Democrats Defend Them	Could Not Determine	Political/Economy	Unclear
5	12/5/19	China Repeats US Must Reduce Tariffs For "Phase One" Trade Deal	True	Political/Economy	Neutral
5	12/9/19	Biden Denies Wrongdoing in Ukraine During Testy Interview	True	Political/Economy	Conservative
7	12/10/19	Stressed to the Max? Deep Sleep Can Rewire the Anxious Brain	True	Science	Neutral
8	12/11/19	Since Feeding the Homeless is Illegal, Activists Carry AR-15s to Give Out Food, Supplies	False/Misleading	Political/Economy	Conservative
9	1/6/20	Senate Republican Eyes Rule Change to Kick Start Trump Impeachment Trial	True	Political/Economy	Neutral
10	1/7/20	Iran Evaluating 13 Retaliation Scenarios To Inflict "Historic Nightmare" On US	True	Political/Economy	Conservative

A.2 Studies 2 and 3

Table S6: Headlines for Articles Chosen from the Low Quality Liberal News Stream in Studies 2 and 3

	Date	Headline	Modal Fact Checker Rating	Topic	Lean of Article
1	11/18/19	Doctor Tells CNN Trump's Walter Reed Medical Visit Was Fishy	False/Misleading	Political/Economy	Liberal
2	11/19/19	Rudy Giuliani has completely berserk meltdown as Feds close in on him	No Mode	Political/Economy	Liberal
3	11/20/19	Lt. Col. Vindman: 'This Is America...Here, Right Matters'	True	Political/Economy	Liberal
4	11/21/19	Sondland's testimony directly implicates Trump, Pence and Pompeo in Ukraine quid pro quo plot	True	Political/Economy	Liberal
5	12/2/19	Senator Announces Impeach Vote Hint That Has Trump Fuming	No Mode	Political/Economy	Liberal
6	12/3/19	The sealed "Indictment A" that Donald Trump needs to worry about more than ever	False/Misleading	Political/Economy	Liberal
7	12/4/19	Devin Nunes Shamelessly Lies When Hannity Asks About Lev Parnas	False/Misleading	Political/Economy	Liberal
8	12/5/19	Trump caught by reporters patting himself on back for insulting Justin Trudeau	True	Political/Economy	Liberal
9	12/9/19	Ex-Intel Slam Trump For Sucking Up To Saudis After Navy Shooting	True	Political/Economy	Liberal
10	12/10/19	Nancy Pelosi knows something we don't	False/Misleading	Political/Economy	Neutral
11	12/11/19	Tucker Carlson's White Power Hour Guest: AOC's District Is The 'Least American'	True	Political/Economy	Liberal
12	12/12/19	Bush's ethics chief: Trumps are an "organized crime family," we need to "go after all of them"	True	Political/Economy	Liberal
13	12/16/19	Trump Attacks Congresswoman For Not Having His Back	True	Political/Economy	Liberal
14	12/17/19	Donald Trump caught retweeting bizarre fake account	No Mode	Political/Economy	Liberal
15	12/18/19	No Punches Pulled In Climate-Themed Campaign Ad	True	Political/Economy	Liberal
16	12/19/19	Shaken Trump vows Democrats will see backlash at "the box office" after impeachment verdict	True	Political/Economy	Liberal
17	1/6/20	Schiff Hammers President & GOP Over Impeachment Trial Obstruction	True	Political/Economy	Liberal
18	1/7/20	Everything is falling apart for Donald Trump in real time	True	Political/Economy	Liberal
19	1/8/20	Trump bewilders nation by tweeting "all is well" and "so far so good" after Iran's missile strike	True	Political/Economy	Liberal
20	1/9/20	John Bolton Will Testify If Subpoenaed, So Why Aren't House Dems Doing That?	No Mode	Political/Economy	Liberal
21	1/13/20	New Trump Approval Poll Released Confirms Massive 2020 Blue Wave	False/Misleading	Political/Economy	Liberal
22	1/14/20	Donald Trump's GOP Senate allies have just been backed into a no-win corner	No Mode	Political/Economy	Liberal
23	1/15/20	Newly released texts from Giuliani collaborator appear to show them stalking Amb. Yovanovich	True	Political/Economy	Liberal
24	1/21/20	Even C-SPAN Is Cut Off From Covering Senate Impeachment Trial	True	Political/Economy	Liberal
25	1/22/20	Schiff Opening Impeachment Trial Statement To Go Down In History	True	Political/Economy	Liberal
26	1/23/20	Donald Trump just screwed up and blew a gaping hole in his own impeachment trial strategy	No Mode	Political/Economy	Liberal
27	1/27/20	Damning potential John Bolton Ukraine impeachment testimony revealed in early leak of book draft	True	Political/Economy	Liberal
28	1/28/20	Joni Ernst Gives Away The Ballgame On Joe Biden	No Mode	Political/Economy	Liberal
29	2/4/20	Donald Trump's sham acquittal is already blowing up in Senate Republicans' faces	No Mode	Political/Economy	Liberal
30	2/5/20	Susan Collins Betrays The Country With Vote To Acquit	True	Political/Economy	Liberal
31	2/6/20	Jennifer Granholm Catches Rick Santorum Shamelessly Lying About Pre-Existing Conditions	False/Misleading	Political/Economy	Liberal

Table S7: Headlines for Articles Chosen from the Low Quality Conservative News Stream in Studies 2 and 3

	Date	Headline	Modal Fact Checker Rating	Topic	Lean of Article
1	11/18/19	KANYE WEST AND HIS SUNDAY SERVICE SHOW PERFORM WITH ILLUMINATI ALL-SEEING EYE OF HORUS STAGE SET AT LAKEWOOD CHURCH TO SOLD OUT CROWD	False/Misleading	Human Interest	Unclear
2	11/19/19	Schiff Named in WH Official's Defamation Lawsuit, Leaked Lies To Politico To Push Impeachment	False/Misleading	Political/Economy	Conservative
3	11/20/19	No Shots Fired! Citizen with a Gun Ends Gunman's Attack at Oklahoma Walmart	False/Misleading	Political/Economy	Conservative
4	11/21/19	Indictment Against Head Of Burisma Reveals 'Hunter Biden Was Receiving Payments From Money Raised Through Criminal Means, Siphoned, Laundered From Ukraine'	False/Misleading	Political/Economy	Conservative
5	12/2/19	Montana Gov. Bullock Drops Out Of 2020 Presidential Race	True	Political/Economy	Neutral
6	12/3/19	Donald Trump SLAMS Corbyn's NHS lies 'We want nothing to do with it!'	False/Misleading	Political/Economy	Neutral
7	12/4/19	In 2018, 86% of Those Arrested for Violent Crime in Los Angeles Were Non-White (5% Were White): the City Is 28% White	False/Misleading	Political/Economy	Conservative
8	12/5/19	DING! DING! DING! First Muslim woman elected to Pennsylvania House of Representatives has been ARRESTED for stealing $500,000 from a charity	True	Political/Economy	Conservative
9	12/9/19	NEVER TRUMPER RICK WILSON SUGGESTS PUTTING ANTI-VAXXERS IN "RE-EDUCATION CAMPS"	True	Political/Economy	Conservative
10	12/10/19	Breaking: Ukrainian Official Reveals Six Criminal Cases Opened In Ukraine Involving The Bidens	False/Misleading	Political/Economy	Conservative
11	12/11/19	Ukraine Advisor Disputes Key Point In Impeachment Testimony — Is This Bad News For Democrats?	False/Misleading	Political/Economy	Conservative
12	12/12/19	NYC's De Blasio Deports Thousands of Homeless Families Across America	False/Misleading	Political/Economy	Conservative
13	12/16/19	Trans Activists Target Olympic Cyclist Inga Thompson For Saying Women Shouldn't Have To Compete With Biological Men	False/Misleading	Human Interest	Conservative
14	12/17/19	Back Home In Pelosi's San Francisco: Homeless Drug Addicts Are Now Taking Dumps In The Supermarket Aisles	False/Misleading	Political/Economy	Conservative
15	12/18/19	Video of the Day: Dem Rep Raskin thanks Congressman helping form rules for sham impeachment of Trump who was impeached for bribery	False/Misleading	Political/Economy	Conservative
16	12/19/19	These Democrats Voted AGAINST Impeaching Trump	False/Misleading	Political/Economy	Conservative
17	1/6/20	NEARLY 200 PEOPLE ARRESTED ACROSS AUSTRALIA FOR DELIBERATELY STARTING BUSHFIRES	False/Misleading	Science	Conservative
18	1/7/20	Iran stampede: '35 dead' and dozens injured after huge crush at Qassem Soleimani funeral	True	Political/Economy	Neutral
19	1/8/20	Muslim Teen Accused Of Starting Aussie Grass Fire Laughs As He Leaves Court On Tuesday	False/Misleading	Science	Conservative
20	1/9/20	Third busiest abortion facility in Massachusetts could soon shut its doors	True	Political/Economy	Conservative

Table S8: Headlines for Articles Chosen from the Low Quality Conservative News Stream in Studies 2 and 3 (Continued)

	Date	Headline	Modal Fact Checker Rating	Topic	Lean of Article
21	1/13/20	Why Are Volcanoes All Over The Globe Suddenly Shooting Giant Clouds Of Ash Miles Into The Air?	False/Misleading	Science	Neutral
22	1/14/20	Wisconsin Judge Orders Up to 209,000 Listings Purged from Voter Rolls — Finds 3 in Contempt, Orders Fines for Delay	True	Political/Economy	Conservative
23	1/15/20	Bloomberg Draws Paltry Crowd Of 45 At Heavily Advertised Rally	Could Not Determine	Political/Economy	Conservative
24	1/21/20	Pentagon bans Bible verses on dog tags, while Pres. Trump upholds right to pray in public schools	False/Misleading	Political/Economy	Conservative
25	1/22/20	LEAKED FRENCH INTERNAL INTELLIGENCE REPORT CLAIMS 150 NEIGHBORHOODS ‘HELD’ BY RADICAL ISLAMISTS	No Mode	Political/Economy	Conservative
26	1/23/20	Coronavirus outbreak: China seals off SECOND major city - 18m people on lockdown	True	Science	Neutral
27	1/27/20	Lawmakers Pushing to Make Michigan a 2nd Amendment Sanctuary STATE	True	Political/Economy	Conservative
28	1/28/20	Holy Moses! More Than 175,000 Tickets Requested To See President Trump In New Jersey — Supporters Line Up 48 Hours Early	False/Misleading	Political/Economy	Conservative
29	2/4/20	Ilhan Omar’s Dirty Money Hustle Blows Wide Open, Reports Say She Gave 40% Of Her Campaign Spending Went To Loverboy’s Firm	False/Misleading	Political/Economy	Conservative
30	2/5/20	DEMS RELEASE ONLY 62% OF IOWA CAUCUS RESULTS — JUST ENOUGH TO HAVE ‘MAYOR CHEAT’ IN THE LEAD	False/Misleading	Political/Economy	Conservative
31	2/6/20	John Kerry Says That The ENTIRE Obama Admin Was Trying To Get Rid Of The Burisma Prosecutor	No Mode	Political/Economy	Conservative

Table S9: Headlines for Articles Chosen from the Mainstream Conservative News Stream in Studies 2 and 3

	Date	Headline	Modal Fact Checker Rating	Topic	Lean of Article
1	11/18/19	Hyundai launches car with a roof-based solar charging system	True	Science	Neutral
2	11/19/19	Pelosi: Trump 's Actions 'Worse' Than Nixon	True	Political/Economy	Neutral
3	11/20/19	Key impeachment witness dodges GOP questions to protect whistleblower	True	Political/Economy	Neutral
4	11/21/19	Smollet Claims He Suffered 'Extreme Emotional Distress' in Malicious Prosecution Lawsuit Against Chicago	True	Human Interest	Neutral
5	12/2/19	'F–K WHITE PEOPLE' graffiti found outside Queens home	True	Human Interest	Neutral
6	12/3/19	Marine veteran turned congressional candidate calls Kaepernick a 'national disgrace'	True	Political/Economy	Conservative
7	12/4/19	Devin Nunes slaps CNN with $435 million defamation lawsuit	True	Political/Economy	Neutral
8	12/5/19	Angry Melania Slams Impeachment Witness for Joking About Son	True	Political/Economy	Conservative
9	12/9/19	Walmart apologizes for sweater featuring Santa with cocaine	True	Human Interest	Neutral
10	12/10/19	Joe Biden Claims No One Told Him About Potential Conflict of Interest With Hunter's Job at Burisma	True	Political/Economy	Conservative
11	12/11/19	House Democrats announce articles of impeachment against Trump: Abuse of power, obstruction of Congress	True	Political/Economy	Neutral
12	12/12/19	Pastors, worship leaders pray for Trump in Oval Office amid impeachment fight	True	Political/Economy	Conservative
13	12/16/19	I was wrong': James Comey admits 'real sloppiness' in Russia probe	True	Political/Economy	Unclear
14	12/17/19	Schiff Says He Would Vote to Impeach Obama If He Engaged in Similar Conduct	True	Political/Economy	Neutral
15	12/18/19	Teen Karol Sanchez staged her own Bronx kidnapping: police sources	True	Human Interest	Neutral
16	12/19/19	President Trump is impeached in a historic vote by the House, will face trial in the Senate	True	Political/Economy	Neutral
17	1/6/20	Ricky Gervais blasts Hollywood figures as unprincipled, ignorant at Golden Globes	True	Human Interest	Neutral
18	1/7/20	Pelosi Says the House Will Vote on a Resolution to Limit Trump's Military Actions Regarding Iran	True	Political/Economy	Neutral
19	1/8/20	Climate Change? Turns Out Two Dozen Arrested for Setting Australia's Fires	False/Misleading	Science	Conservative
20	1/9/20	Cardi B bashes Trump, says she's seeking Nigerian citizenship amid tensions with Iran	True	Political/Economy	Neutral
21	1/13/20	Bill Gates: My $109 billion net worth shows the economy is not fair	True	Political/Economy	Neutral
22	1/14/20	Trump, first lady cheered at national championship game	True	Political/Economy	Neutral
23	1/15/20	President Trump Gets Thunderous Applause at Clemson and LSU National Championship Game	True	Political/Economy	Conservative
24	1/21/20	Virginia's Capitol flooded with gun rights activists for Second Amendment rally	True	Political/Economy	Conservative
25	1/22/20	CDC confirms first US case of coronavirus that has killed 9 in China	True	Science	Neutral
26	1/23/20	Three US firefighters killed in plane crash while battling wildfires in Australia	True	Science	Neutral
27	1/27/20	Coronavirus may have originated in lab linked to China's biowarfare program	No Mode	Science	Neutral
28	1/28/20	Dershowitz calls out House Dems in Trump's Senate impeachment trial after Bolton shock waves	True	Political/Economy	Conservative
29	2/4/20	Democratic White House Race off to Messy Start as 'Inconsistencies' Delay Iowa Results	True	Political/Economy	Neutral
30	2/5/20	Macy's to close 125 stores, cut 2,000 corporate jobs, in hunt for growth	True	Political/Economy	Neutral
31	2/6/20	Trump acquitted on all charges in Senate impeachment trial	True	Political/Economy	Neutral

Table S10: Headlines for Articles Chosen from the Mainstream Liberal News Stream in Studies 2 and 3

	Date	Headline	Modal Fact Checker Rating	Topic	Lean of Article
1	11/18/19	10 shot, four killed at family gathering in Fresno, California	True	Human Interest	Neutral
2	11/19/19	Kanye West calls himself ”greatest artist that God has ever created” during Joel Osteen service	True	Human Interest	Neutral
3	11/20/19	Woman Saves Scorched Koala From Bushfire With Shirt Off Her Own Back	True	Science	Neutral
4	11/21/19	Almaas Elman, Somali-Canadian Activist, Is Shot Dead in Mogadishu	True	Political/Economy	Neutral
5	12/2/19	White House will not participate in Wednesday’s impeachment hearing	True	Political/Economy	Neutral
6	12/3/19	Duncan Hunter To Plead Guilty In Campaign Finance Case He Called ’Witch Hunt’	True	Political/Economy	Neutral
7	12/4/19	Kamala Harris Dropping Out Of Presidential Race	True	Political/Economy	Neutral
8	12/5/19	‘He Showed Us Life’: Japanese Doctor Who Brought Water to Afghans Is Killed	True	Human Interest	Neutral
9	12/9/19	Caroll Spinney, legendary ’Sesame Street’ puppeteer of Big Bird, dies at 85	True	Human Interest	Neutral
10	12/10/19	Megan Rapinoe is Sports Illustrated’s Sportsperson of the Year, only the fourth woman chosen alone	True	Human Interest	Neutral
11	12/11/19	Police Chief Tears Into Ted Cruz, McConnell For Caring More About NRA Than Gun Victims	True	Political/Economy	Neutral
12	12/12/19	Donald Trump Jr killed rare endangered sheep in Mongolia with special permit	True	Other	Liberal
13	12/16/19	Black Women Now Hold Crowns in 5 Major Beauty Pageants	True	Human Interest	Neutral
14	12/17/19	Barack Obama: Women Ruling All Nations Would Improve ’Just About Everything’	True	Political/Economy	Neutral
15	12/18/19	Police investigating whether teen staged her own kidnapping in Bronx	True	Human Interest	Neutral
16	12/19/19	House impeaches Trump for abuse of power and obstruction in historic rebuke	True	Political/Economy	Neutral
17	1/6/20	Mike Pence Slammed After Falsely Linking Qassem Soleimani To 9/11	True	Political/Economy	Liberal
18	1/7/20	Pentagon Rules Out Striking Iranian Cultural Sites, Contradicting Trump	True	Political/Economy	Liberal
19	1/8/20	All is well,’ Trump tweets after Iran targets U.S. forces in missile attack in Iraq	True	Political/Economy	Neutral
20	1/9/20	Ruth Bader Ginsburg says she is cancer-free	True	Political/Economy	Neutral
21	1/13/20	Serena Williams wins first title in 3 years — and donates prize money to Australia wildfire relief	True	Human Interest	Neutral
22	1/14/20	The first Obama-backed documentary receives an Oscar nomination	True	Human Interest	Neutral
23	1/15/20	More than 50 injured after Delta jet dumps fuel on L.A. schools during midair emergency	True	Human Interest	Neutral
24	1/21/20	Katie Sowers Is The First Female And Openly Gay Person To Coach In A Super Bowl	True	Human Interest	Neutral
25	1/22/20	Weather service issues alert for falling iguanas as temperatures drop in Florida	True	Science	Neutral
26	1/23/20	Half of Americans don’t know 6m Jews were killed in Holocaust, survey says	True	Political/Economy	Neutral
27	1/27/20	Kobe Bryant’s Daughter Gianna, 13, Dead Alongside Father in Calabasas Helicopter Crash	True	Human Interest	Neutral
28	1/28/20	Today really hurts’: Families, friends remember those who died in Kobe Bryant crash	True	Human Interest	Neutral
29	2/4/20	State of the Union 2020: Trump addresses nation just before expected acquittal by Senate	True	Political/Economy	Neutral
30	2/5/20	Nancy Pelosi rips up copy of State of the Union speech from Trump	True	Political/Economy	Neutral
31	2/6/20	Kirk Douglas, Hollywood legend and star of Spartacus, dies aged 103	True	Human Interest	Neutral

Table S11: Headlines for Articles Chosen from the Low Quality Unclear News Stream in Studies 2 and 3

	Date	Headline	Modal Fact Checker Rating	Topic	Lean of Article
1	11/18/19	Family Facing Jail for Living in RV on Their Own Property to Repair Home After Fire	True	Human Interest	Conservative
2	11/19/19	Shooter Commits Suicide After Being Confronted by Armed Citizen at OK Walmart	False/Misleading	Human Interest	Neutral
3	11/20/19	Pounds lost doesn't mean FAT lost: You CAN lose up to 2 pounds of fat a month – but it takes consistency and patience	False/Misleading	Science	Neutral
4	11/21/19	Ukrainian MP Claims $7.4 Billion Obama-Linked Laundering, Puts Biden Group Take At $16.5 Million	False/Misleading	Political/Economy	Conservative
5	12/2/19	Bestselling Novelist Who Wrote About Vaccine Industry Deception Found Dead	False/Misleading	Human Interest	Unclear
6	12/3/19	Americans Bought Enough Guns on Black Friday to Arm the Marine Corps – Yet Again!	True	Political/Economy	Unclear
7	12/4/19	Ukrainian Neo-Nazis Help Out at Hong Kong Riots, Pan-Democrats Defend Them	Could Not Determine	Political/Economy	Unclear
8	12/5/19	China Repeats US Must Reduce Tariffs For "Phase One" Trade Deal	True	Political/Economy	Neutral
9	12/9/19	Biden Denies Wrongdoing in Ukraine During Testy Interview	True	Political/Economy	Conservative
10	12/10/19	Stressed to the Max? Deep Sleep Can Rewire the Anxious Brain	True	Science	Neutral
11	12/11/19	Since Feeding the Homeless is Illegal, Activists Carry AR-15s to Give Out Food, Supplies	False/Misleading	Political/Economy	Conservative
12	12/12/19	Russia's Only Aircraft Carrier Has Erupted In Flames	True	Political/Economy	Neutral
13	12/16/19	Trump Poised This Week to Become Third U.S. President Impeached	True	Political/Economy	Neutral
14	12/17/19	Kansas City Makes Public Transportation Free, Become The First Major City In The U.S. To Make This Progressive Change	No Mode	Political/Economy	Liberal
15	12/18/19	Wall Street Journal Investigation Finds Amazon.com Selling Dumpster Trash Food & Supplements As New	No Mode	Science	Neutral
16	12/19/19	UN Peacekeepers Fathered Hundreds of Babies With Girls in Haiti as Young as 11	True	Political/Economy	Conservative
17	1/6/20	Senate Republican Eyes Rule Change to Kick Start Trump Impeachment Trial	True	Political/Economy	Neutral
18	1/7/20	Iran Evaluating 13 Retaliation Scenarios To Inflict "Historic Nightmare" On US	True	Political/Economy	Conservative
19	1/8/20	Key Brain Region Smaller in Birth Control Pill User	True	Science	Neutral
20	1/9/20	The US Military Pollutes More 140 Countries Combined	True	Science	Liberal

Table S12: Headlines for Articles Chosen from the Low Quality Unclear News Stream in Studies 2 and 3 (Continued)

	Date	Headline	Modal Fact Checker Rating	Topic	Lean of Article
21	1/13/20	Alaska man survives three weeks with little food and shelter	True	Human Interest	Neutral
22	1/14/20	Boeing Mocked Lion Air ”Idiots” For Requesting Extra Training For 737 MAX	True	Human Interest	Unclear
23	1/15/20	300 Vultures Occupy Border Patrol Tower, Covering It With “Corrosive” Feces & Vomit	True	Human Interest	Neutral
24	1/21/20	PUNISHING ECONOMY: San Fran’s Democrat tyrants double down on closed businesses, taxing landlords for leaving stores vacant	False/Misleading	Political/Economy	Conservative
25	1/22/20	Another Supposedly Authentic Photo Of A UFO & The Story Behind It	No Mode	Human Interest	Neutral
26	1/23/20	China Quarantines 3rd City As Wuhan Virus Spreads To Singapore	True	Science	Neutral
27	1/27/20	Nature Science Journal Warned About “Pathogens Escaping” Wuhan Level-4 Biosafety Lab (BSL-4) Before Coronavirus Outbreak	False/Misleading	Science	Unclear
28	1/28/20	Death Tolls Rises to 106 as 1,000 Americans Try to Evacuate From Coronavirus-Infected Wuhan	True	Science	Neutral
29	2/4/20	The Coronavirus Was Engineered By Scientists In A Lab Using Well Documented Genetic Engineering Vectors That Leave Behind A “Fingerprint”	False/Misleading	Science	Unclear
30	2/5/20	Earth is About to Enter a 30-Year ‘Mini Ice Age’ as the Sun Hibernates, Scientist Warns	False/Misleading	Science	Unclear
31	2/6/20	The lies we are being told about the Coronavirus	False/Misleading	Science	Conservative

A.3 Study 4

Table S13: Headlines for Articles Chosen from the Low Quality Liberal News Stream in Study 4

	Date	Headline	Modal Fact Checker Rating	Topic	Lean of Article
1	5/27/20	Only 1 state has met the federal government's criteria for reopening	False/Misleading	Science	Neutral
2	6/1/20	Republican Voters Don't Expect Trump To Mourn, Because They Gave Up On Empathy Long Ago	Could Not Determine	Political/Economy	Liberal
3	6/3/20	Communities Of Color Have Been Hit Hardest By COVID-19. Now Is The Time To Fix That	True	Political/Economy	Liberal
4	6/8/20	CDC: More Americans Drinking Cleaning Products Than Ever Before	No Mode	Science	Liberal
5	6/10/20	New Zealand Is COVID-Free; Prime Minister 'Did A Little Dance'	True	Science	Neutral
6	6/15/20	Larry Kudlow: Attendees Of Trump's Tulsa Rally Should 'Probably' Wear Masks	True	Political/Economy	Liberal
7	6/17/20	Trump Touts Aids Vaccine That Does Not Exist During Tuesday Meltdown	True	Political/Economy	Liberal
8	6/22/20	COVID Infection Spike In At Least 23 States, More Young People Testing Positive	True	Other	Liberal

Table S14: Headlines for Articles Chosen from the Low Quality Conservative News Stream in Study 4

	Date	Headline	Modal Fact Checker Rating	Topic	Lean of Article
1	5/27/20	Like the Soviet-Styled KGB,' Armed Police Sent To Shut Down Black Baptist Church, Pastor Says	False/Misleading	Political/Economy	Conservative
2	6/1/20	Trump pulls U.S. out of World Health Organization: Is the U.N. next?	False/Misleading	Political/Economy	Conservative
3	6/3/20	Leftie Governor Cooper Kills RNC Convention in Charlotte Due to COVID-19 Ñ Then Goes and Marches with Leftist Mob in Street (VIDEO)	False/Misleading	Political/Economy	Conservative
4	6/8/20	Forced' vaccinations will control your life, warns religious-liberty group	False/Misleading	Science	Neutral
5	6/10/20	Task Force: Expect a Spike in COVID Thanks to Protesters and Rioters	False/Misleading	Other	Conservative
6	6/15/20	CHINA LOCKS DOWN TEN MORE BEIJING NEIGHBORHOODS OVER NEW COVID-19 OUTBREAK AT WHOLESALE MARKET	True	Other	Neutral
7	6/17/20	Thousands Gather in NYC for 'Black Trans Lives Matter' Protest on Same Day Cuomo Threatens Businesses	True	Political/Economy	Conservative
8	6/22/20	Frightened' doctor warns against using hand dryers as they will spark coronavirus spike	True	Science	Neutral

Table S15: Headlines for Articles Chosen from the Low Quality Non-Partisan News Stream in Study 4

	Date	Headline	Modal Fact Checker Rating	Topic	Lean of Article
1	5/27/20	Your ÒImmunity PassportÓ Future Begins To Materialize As Airlines Call For Digital ID Tracking Systems	False/Misleading	Science	Neutral
2	6/1/20	German Official Leaks Report Denouncing COVID-19 As ”A Global False Alarm”	False/Misleading	Political/Economy	Conservative
3	6/3/20	CONTACT TRACING IN THE CIRCUS OF ROBOTS	False/Misleading	Other	Conservative
4	6/8/20	Bill Gates, The CDC, Fauci And Birx Now Totally Silent About The Mass Race Riots Across America Proves The COVID-19 Lockdown Was A Total Scam	False/Misleading	Other	Conservative
5	6/10/20	WHO Data Suggests ItÕs ÔVery RareÕ For COVID-19 To Spread Through Asymptomatic People	False/Misleading	Science	Conservative
6	6/15/20	Chinese Scientist, Escorted Out Of Canadian Biolab, Sent Deadly Viruses To Wuhan	False/Misleading	Science	Neutral
7	6/17/20	FOX: Cobb County man tests positive and negative for COVID-19 just hours apart	True	Science	Neutral
8	6/22/20	Dr. Meryl Nass Discovers Hydroxychloroquine Experiments Were Designed to Kill COVID Patients How Many Were Murdered?	False/Misleading	Science	Unclear

Table S16: Headlines for Articles Chosen from the Mainstream Conservative News Stream in Study 4

	Date	Headline	Modal Fact Checker Rating	Topic	Lean of Article
1	5/27/20	House Republicans sue Pelosi in bid to stop proxy voting amid coronavirus concerns	True	Political/Economy	Neutral
2	6/1/20	Second wave of coronavirus infections could cause a worse economic disaster, experts warn	True	Political/Economy	Neutral
3	6/3/20	Hope: Top Italian Doctors Say COVID-19 is Losing Viral Potency, Becoming Less Deadly	True	Science	Neutral
4	6/8/20	As states reopen and protests rage, the coronavirus lays the foundation for a nasty second wave of infections this fall	True	Science	Neutral
5	6/10/20	Texas sees record number of coronavirus hospitalizations after state reopens	True	Other	Neutral
6	6/15/20	US coronavirus deaths could double, hit 200,000 by September: report	True	Science	Neutral
7	6/17/20	Scientists hail dexamethasone as Ômajor breakthroughÕ in treating coronavirus	True	Science	Neutral
8	6/22/20	U.S. reports more than 30,000 coronavirus cases two days straight, the highest number since May 1	True	Other	Neutral

Table S17: Headlines for Articles Chosen from the Mainstream Liberal News Stream in Study 4

	Date	Headline	Modal Fact Checker Rating	Topic	Lean of Article
1	5/27/20	Fauci says he wears a mask to be a symbol of what 'you should be doing'	True	Science	Neutral
2	6/1/20	How a decade of privatisation and cuts exposed England to coronavirus	True	Political/Economy	Neutral
3	6/3/20	Trump slams North Carolina and says he's moving GOP convention elsewhere	True	Political/Economy	Neutral
4	6/8/20	With no active Covid-19 cases, New Zealand is lifting almost all its coronavirus restrictions	True	Other	Neutral
5	6/10/20	Majority of UK theatres and music venues 'face permanent shutdown'	True	Political/Economy	Neutral
6	6/15/20	Florida sees 2 consecutive days of 2,000-plus new COVID-19 cases as more beaches reopen	True	Other	Neutral
7	6/17/20	Trump Claims COVID-19 Will Go Away And That An AIDS Vaccine Exists. It Doesn't.	True	Political/Economy	Liberal
8	6/22/20	Trump trade adviser: Rally comment on reducing Covid testing was just a joke	True	Political/Economy	Liberal

A.4 Study 5

Table S18: Headlines for Articles Chosen from the Low Quality Conservative News Stream in Study 5

	Date	Headline	Modal Fact Checker Rating	Topic	Lean of Article
1	7/19/21	AZ State Senator Demands Biden Electors Be Recalled, New Election Be Held After Shocking Audit Revelations	False/Misleading	Politics	Conservative
2	7/20/21	'Know Your History, Coward': UFC Star Calls Out Colin Kaepernick For Support Of Fidel Castro	True	No Mode	Conservative
3	7/21/21	Democrats Add Funding Of 'Civilian Climate Corps' To Infrastructure Bill	True	Politics	Conservative
4	7/22/21	GOP Reps. Demand Explanation For Why Capitol Protesters Are Being Jailed When BLM Protesters Were Not	No Mode	Politics	Conservative
5	7/25/21	Enthusiasm for Trump's rally in Arizona dwarfs President Biden's town hall just days earlier	False/Misleading	Politics	Conservative
6	7/26/21	Ashli Babbitt's Mom: Nancy Pelosi Orchestrated the Killing of My Daughter	False/Misleading	Politics	Conservative
7	7/27/21	'They Tortured Me': Officer Michael Fanone Calls Jan. 6 Rioters 'Terrorists'	True	Politics	Unclear
8	7/28/21	Greg Abbott Orders Texas National Guard To Begin Arresting Illegal Immigrants	True	Politics	Conservative
9	10/28/21	GOP Rep. Duncan Enrages Democrat By Wearing 'Let's Go Brandon' Mask On House Floor	True	Politics	Conservative
10	11/1/21	Shock NBC Poll Shows Americans Have 'Lost Their Confidence' In Biden, Chuck Todd Says	True	Politics	Conservative
11	11/3/21	Foreign News Media Laughs at Joe Biden: He 'Needs a Retirement Home and a Warm Bowl of Soup'	False/Misleading	Politics	Conservative
12	11/8/21	Indictment shows it was Hillary who colluded with Russia	False/Misleading	Politics	Conservative

Table S19: Headlines for Articles Chosen from the Low Quality Liberal News Stream in Study 5

	Date	Headline	Modal Fact Checker Rating	Topic	Lean of Article
1	7/19/21	Miserable Trump throws fit because a British golf tournament isn't held at his course	True	Politics	Liberal
2	7/20/21	Bans On Critical Race Theory Will Have A Chilling Effect On Educators	No Mode	Politics	Liberal
3	7/21/21	Looks like New York prosecutors have a witness directly incriminating Donald Trump	False/Misleading	Politics	Liberal
4	7/22/21	Liz Cheney Emasculates Kevin McCarthy For His Jan 6 Nonsense	True	Politics	Liberal
5	7/25/21	Rand Paul is left speechless after Dr. Fauci tears into him for lying in Senate hearing	False/Misleading	No Mode	Liberal
6	7/26/21	Newsmax Jackass Roots For 'Woke' Team USA To Lose	True	Human Interest	Liberal
7	7/27/21	Matt Gaetz and Marjorie Taylor Greene get run out of their own protest rally	False/Misleading	Politics	Liberal
8	7/28/21	Capitol Police Chief Destroys GOP Narrative After Jan. 6 Hearing	True	Politics	Liberal
9	10/28/21	Biden Denies Trump Exec Privilege & Releases Treasure-Trove Of Docs	True	Politics	Liberal
10	11/1/21	Private Jet Flying Insurrectionist Smacked Down By DOJ Over Jan 6	No Mode	Politics	Liberal
11	11/3/21	Ivermectin Study Retracted After Data Found To Be Completely Wrong	True	Science	Liberal
12	11/8/21	Liz Cheney Appears On 'Fox Sunday' To Hand Trump His Ass	True	Politics	Liberal

Table S20: Headlines for Articles Chosen from the Low Quality Non-Partisan News Stream in Study 5

	Date	Headline	Modal Fact Checker Rating	Topic	Lean of Article
1	7/19/21	”This Is Worrying Me Quite A Bit”: mRNA Vaccine Inventor Shares Viral Thread Showing COVID Surge In Most-Vaxxed Countries	False/Misleading	Science	Unclear
2	7/20/21	Texas Democrats’ DC Trip To Block Voting Bill Expected To Cost Around $1.5 Million	True	Politics	Unclear
3	7/21/21	Jeff Bezos Thanks Amazon Workers And Customers For Paying For His Flight To Space	True	Human Interest	Unclear
4	7/22/21	“Zero COVID” Catastrophe: Participating Nations See New Records Across the Board	False/Misleading	Science	Unclear
5	7/25/21	Chicago Chamber Of Commerce Rages As Average Unemployed Illinoisan Parent Earns $35/Hour Sitting On The Couch	False/Misleading	Economy	Conservative
6	7/26/21	High Schooler Raises $12K to Help Homeless Veterans Across the Country Get Off the Streets	True	Human Interest	Neutral
7	7/27/21	REPORT: Democrats Running In Rural Areas Are Keeping Their Party Identification Out Of Their Ads	No Mode	Politics	Conservative
8	7/28/21	REPORT: lhan Omar cites rising COVID cases to make demand of Biden. Pelosi says	True	Politics	Unclear
9	10/28/21	NIH Director Shredded Over Risky Research In Wuhan After CNN Interview Goes Sideways	False/Misleading	Science	Conservative
10	11/1/21	U.S. faces engineered famine as COVID lockdowns and vax mandates could lead to widespread hunger, unrest this winter	False/Misleading	No Mode	Conservative
11	11/3/21	’Falsified Data’: Pfizer Vaccine Trial Had Major Flaws, Whistleblower Tells Peer-Reviewed Journal	False/Misleading	Science	Conservative
12	11/8/21	Biden Gang Reportedly Running Two Secret Lists Used to Prevent Outspoken Conservatives from Owning Guns – Laura Loomer Speaks Out on This Injustice	False/Misleading	Politics	Conservative

Table S21: Headlines for Articles Chosen from the Mainstream Conservative News Stream in Study 5

	Date	Headline	Modal Fact Checker Rating	Topic	Lean of Article
1	7/19/21	Majority of Arizona Republicans believe election audit will show Trump won, poll shows	True	Politics	Unclear
2	7/20/21	Sixth Texas Dem comes down with COVID-19 and is isolating: report	True	Politics	Conservative
3	7/21/21	23-Year-Old Ex-Trump Staffer Running for Congress in New Hampshire	True	Politics	Conservative
4	7/22/21	Video shows would-be LA robbery victim shoot his assailants	True	Human Interest	Unclear
5	7/25/21	Banks: Pelosi Doesn't Want 'Tough Questions' Because She's Responsible for Breakdown of Security on Jan. 6'	False/Misleading	Politics	Conservative
6	7/26/21	Trump Rejects 'Fake' Jan. 6 Panel: 'Will Nancy Investigate Herself?'	False/Misleading	Politics	Conservative
7	7/27/21	'Running against a movement': Eric Adams declares war on AOC's socialists	True	Politics	Unclear
8	7/28/21	Olympian Caeleb Dressel tears up on gold medal stand while national anthem plays	True	Human Interest	Unclear
9	10/28/21	Rep. Marjorie Taylor Greene bought shares of Trump SPAC Digital World Acquisition as stock skyrocketed	True	Politics	Neutral
10	11/1/21	AMA Document: Doctors Should Use Language 'Inspired by Critical Race Theory'	True	Science	Unclear
11	11/3/21	McAuliffe Concedes Virginia Governor's Race	True	Politics	Neutral
12	11/8/21	Nearly half of voters say Biden worse president than expected, most don't want to see him run again: poll	True	Politics	Conservative

Table S22: Headlines for Articles Chosen from the Mainstream Liberal News Stream in Study 5

	Date	Headline	Modal Fact Checker Rating	Topic	Lean of Article
1	7/19/21	Kara Eaker, U.S. women's gymnastics alternate, tests positive for Covid days before Tokyo Olympics	True	Human Interest	Neutral
2	7/20/21	Twitter suspends Rep. Marjorie Taylor Greene for spreading COVID-19 misinformation	True	Politics	Neutral
3	7/21/21	U.S. stunned by Sweden 3-0 in women's soccer at Olympics: "Not the start we wanted"	True	Human Interest	Neutral
4	7/22/21	Alabama city leader who used n-word in council meeting says he won't apologize and might run for mayor	True	No Mode	Neutral
5	7/25/21	Tom Daley 'incredibly proud to say I am gay and an Olympic champion'	True	Human Interest	Neutral
6	7/26/21	Parkland Survivor Says QAnon Convinced His Dad Shooting Was A Hoax: Report	Could Not Determine	Human Interest	Neutral
7	7/27/21	As Covid cases surge, unvaccinated Americans trigger scorn, resentment from many vaccinated people	True	Human Interest	Neutral
8	7/28/21	Simone Biles says she now realizes she is more than her gymnastics career in emotional tweet	True	Human Interest	Neutral
9	10/28/21	DeVos family was misled by Theranos founder before investing $100 million, rep says	True	Human Interest	Neutral
10	11/1/21	Steve Buscemi Hands Out Candy Dressed As His Own Meme On Halloween	True	Human Interest	Neutral
11	11/3/21	QAnon supporters gather over theory that JFK Jr. will emerge, announce Trump to be reinstated	True	Human Interest	Neutral
12	11/8/21	Aaron Rodgers says he's unvaccinated, takes ivermectin and bashes 'woke mob'	True	Human Interest	Neutral

B Model Results for Figures 1–4 in Main Text

Figure 1a and Figure 1b

Table S23: Results from OLS Regression Results Presented in Figure 1a and 1b (Study 1). This table presents the average treatment effects for linear regression models testing the effect of SOTEN. All effects are estimated using ordinary least squares (OLS) with article fixed effects and standard errors clustered at the individual and article level.

Dependent Variables:	Categorical (Rated as True)	7-Point Ordinal Scale
Model:	(1)	(2)
Variables		
Treatment (Search)	0.0568*	0.1615
	(0.0243)	(0.1061)
Age	-0.0029**	-0.0110***
	(0.0007)	(0.0024)
Ideological Congruence	0.1737**	0.9850***
	(0.0425)	(0.1585)
Education	-0.0066	-0.0130
	(0.0118)	(0.0495)
Gender (Female dummy)	-0.0329	-0.0165
	(0.0226)	(0.0915)
Income	-0.0021	0.0088
	(0.0099)	(0.0349)
Fixed-effects		
Article	Yes	Yes
Fit statistics		
Observations	2,275	2,275
R^2	0.05869	0.07744
Within R^2	0.03110	0.04894

Clustered (Article & Respondent) standard-errors in parentheses
*Signif. Codes: ***: 0.001, **: 0.01, *: 0.05*

Table S24: Results from OLS Regression Results Presented in Figure 1a and 1b (Study 2). This table presents the average treatment effects for linear regression models testing the effect of SOTEN. All effects are estimated using ordinary least squares (OLS) with article fixed effects and standard errors clustered at the individual and article level.

Dependent Variables:	Categorical (Rated as True)	7-Point Ordinal Scale
Model:	(1)	(2)
Variables		
Treatment (Search)	0.0713***	0.2426***
	(0.0159)	(0.0616)
Age	-0.0021*	-0.0033
	(0.0008)	(0.0034)
Ideological Congruence	0.1790***	0.8350***
	(0.0386)	(0.1574)
Education	-0.0138	-0.0061
	(0.0126)	(0.0406)
Gender (Female dummy)	-0.0238	0.0076
	(0.0322)	(0.0911)
Income	0.0032	-0.0253
	(0.0135)	(0.0542)
Fixed-effects		
Article	Yes	Yes
Fit statistics		
Observations	2,020	2,020
R^2	0.11677	0.11984
Within R^2	0.03413	0.04105

Clustered (Article & Respondent) standard-errors in parentheses
*Signif. Codes: ***: 0.001, **: 0.01, *: 0.05*

Table S25: Results from OLS Regression Results Presented in Figure 1a and 1b (Study 3). This table presents the average treatment effects for linear regression models testing the effect of SOTEN. All effects are estimated using ordinary least squares (OLS) with article fixed effects and standard errors clustered at the individual and article level.

Dependent Variables:	Categorical (Rated as True)	7-Point Ordinal Scale
Model:	(1)	(2)
Variables		
Treatment (Search)	0.0662**	0.2322**
	(0.0195)	(0.0743)
Age	-0.0015	-0.0008
	(0.0008)	(0.0026)
Ideological Congruence	0.1912***	0.8511***
	(0.0369)	(0.1387)
Education	0.0089	0.0287
	(0.0097)	(0.0392)
Gender (Female dummy)	-0.0632*	-0.1021
	(0.0245)	(0.0992)
Income	0.0111	0.0134
	(0.0143)	(0.0533)
Fixed-effects		
Article	Yes	Yes
Fit statistics		
Observations	1,964	1,964
R^2	0.10520	0.11288
Within R^2	0.04113	0.04231

Clustered (Article & Respondent) standard-errors in parentheses
*Signif. Codes: ***: 0.001, **: 0.01, *: 0.05*

Table S26: Results from OLS Regression Results Presented in Figure 1a and 1b (Study 4). This table presents the average treatment effects for linear regression models testing the effect of SOTEN. All effects are estimated using ordinary least squares (OLS) with article fixed effects and standard errors clustered at the individual and article level.

Dependent Variables:	Categorical (Rated as True)	7-Point Ordinal Scale
Model:	(1)	(2)
Variables		
Treatment (Search)	0.0674*	0.2617**
	(0.0301)	(0.0774)
Age	-0.0039*	-0.0100
	(0.0013)	(0.0064)
Ideological Congruence	0.1675*	0.7408***
	(0.0554)	(0.1330)
Education	0.0344	-0.0024
	(0.0164)	(0.0804)
Gender (Female dummy)	-0.0281	-0.2139
	(0.0534)	(0.2192)
Income	0.0039	0.0126
	(0.0240)	(0.1168)
Fixed-effects		
Article	Yes	Yes
Fit statistics		
Observations	772	772
R^2	0.09822	0.09963
Within R^2	0.04502	0.03786

Clustered (Article & Respondent) standard-errors in parentheses
*Signif. Codes: ***: 0.001, **: 0.01, *: 0.05*

Figure 2b

Table S27: Results from OLS Regression Results Presented in Figure 2b. This table presents the average treatment effects for linear regression models testing the effect of SOTEN. All effects are estimated using ordinary least squares (OLS) with article fixed effects and standard errors clustered at the individual and article level.

Dependent Variables:	Categorical (Rated as True)	4-Point Ordinal Scale	7-Point Ordinal Scale
Model:	(1)	(2)	(3)
Variables			
Treatment (Search)	0.1068*	0.1595*	0.1792
	(0.0388)	(0.0727)	(0.1344)
Age	0.0006	0.0035*	0.0060
	(0.0009)	(0.0015)	(0.0037)
Ideological Congruence	0.2799***	0.6096***	1.185***
	(0.0388)	(0.0838)	(0.1572)
Education	-0.0059	-0.0400	-0.0482
	(0.0144)	(0.0266)	(0.0539)
Gender (Female dummy)	0.0068	-0.0537	-0.0129
	(0.0279)	(0.0580)	(0.0976)
Income	-0.0058	-0.0295	-0.0440
	(0.0162)	(0.0325)	(0.0613)
Fixed-effects			
Article	Yes	Yes	Yes
Fit statistics			
Observations	1,485	1,485	1,485
R^2	0.12286	0.16222	0.15156
Within R^2	0.07909	0.09227	0.08846

Clustered (Article & Respondent) standard-errors in parentheses
*Signif. Codes: ***: 0.001, **: 0.01, *: 0.05*

Figure 2c

Table S28: Results from OLS Regression Results Presented in Figure 2c (Only Very Reliable News Returned). This table presents the average treatment effects for linear regression models testing the effect of SOTEN. All effects are estimated using ordinary least squares (OLS) with article fixed effects and standard errors clustered at the individual and article level.

Dependent Variables:	Categorical Scale (True = 1)	Ordinal Scale (4-Point)	Ordinal Scale (7-Point)
Model:	(1)	(2)	(3)
Variables			
Treatment (Search)	-0.0068	0.0294	-0.1728
	(0.0727)	(0.1211)	(0.2737)
Age	9.07×10^{-5}	0.0039	0.0081
	(0.0012)	(0.0020)	(0.0052)
Ideological Congruence	0.3126***	0.6470***	1.221***
	(0.0389)	(0.0887)	(0.1483)
Education	0.0035	-0.0312	-0.0183
	(0.0180)	(0.0274)	(0.0585)
Gender (Female dummy)	0.0026	-0.0847	-0.0577
	(0.0281)	(0.0707)	(0.1105)
Income	-0.0097	-0.0440	-0.0420
	(0.0226)	(0.0419)	(0.0852)
Fixed-effects			
Article	Yes	Yes	Yes
Fit statistics			
Observations	940	940	940
R^2	0.13475	0.16525	0.15168
Within R^2	0.07493	0.09156	0.08699

Clustered (Article & Respondent) standard-errors in parentheses
*Signif. Codes: ***: 0.001, **: 0.01, *: 0.05*

Table S29: Results from OLS Regression Results Presented in Figure 2c (Some Unreliable News Returned). This table presents the average treatment effects for linear regression models testing the effect of SOTEN. All effects are estimated using ordinary least squares (OLS) with article fixed effects and standard errors clustered at the individual and article level.

Dependent Variables:	Categorical Scale (True = 1)	Ordinal Scale (4-Point)	Ordinal Scale (7-Point)
Model:	(1)	(2)	(3)
Variables			
Treatment (Search)	0.1416**	0.1968*	0.2762
	(0.0425)	(0.0834)	(0.1490)
Age	9.49×10^{-5}	0.0040	0.0068
	(0.0011)	(0.0020)	(0.0045)
Ideological Congruence	0.3146***	0.6592***	1.258***
	(0.0405)	(0.0789)	(0.1412)
Education	-0.0089	-0.0582*	-0.0698
	(0.0172)	(0.0273)	(0.0593)
Gender (Female dummy)	-0.0179	-0.1123	-0.1182
	(0.0343)	(0.0865)	(0.1321)
Income	-0.0098	-0.0610	-0.0669
	(0.0225)	(0.0427)	(0.0865)
Fixed-effects			
Article	Yes	Yes	Yes
Fit statistics			
Observations	1,027	1,027	1,027
R^2	0.14265	0.17254	0.15964
Within R^2	0.08953	0.10766	0.09852

Clustered (Article & Respondent) standard-errors in parentheses
*Signif. Codes: ***: 0.001, **: 0.01, *: 0.05*

Figure 2d

Table S30: Results from OLS Regression Results Presented in Figure 2d (0-25 Percentage Quartile of News Quality). This table presents the average treatment effects for linear regression models testing the effect of SOTEN. All effects are estimated using ordinary least squares (OLS) with article fixed effects and standard errors clustered at the individual and article level.

Dependent Variables:	Categorical Scale (True = 1)	Ordinal Scale (4-Point)	Ordinal Scale (7-Point)
Model:	(1)	(2)	(3)
Variables			
Treatment (Search)	0.0934*	0.1095	0.0924
	(0.0381)	(0.0776)	(0.1570)
Age	0.0002	0.0042	0.0075
	(0.0011)	(0.0022)	(0.0048)
Ideological Congruence	0.3197***	0.6794***	1.283***
	(0.0426)	(0.0832)	(0.1498)
Education	-0.0050	-0.0489	-0.0633
	(0.0181)	(0.0270)	(0.0585)
Gender (Female dummy)	-0.0253	-0.1148	-0.1538
	(0.0314)	(0.0789)	(0.1247)
Income	-0.0068	-0.0454	-0.0428
	(0.0220)	(0.0448)	(0.0870)
Fixed-effects			
Article	Yes	Yes	Yes
Fit statistics			
Observations	1,006	1,006	1,006
R^2	0.14164	0.17250	0.16143
Within R^2	0.08408	0.10378	0.09707

Clustered (Article & Respondent) standard-errors in parentheses
*Signif. Codes: ***: 0.001, **: 0.01, *: 0.05*

Table S31: Results from OLS Regression Results Presented in Figure 2d (25-50 Percentage Quartile of News Quality). This table presents the average treatment effects for linear regression models testing the effect of SOTEN. All effects are estimated using ordinary least squares (OLS) with article fixed effects and standard errors clustered at the individual and article level.

Dependent Variables:	Categorical Scale (True = 1)	Ordinal Scale (4-Point)	Ordinal Scale (7-Point)
Model:	(1)	(2)	(3)
Variables			
Treatment (Search)	0.1698*	0.2386	0.3939
	(0.0608)	(0.1177)	(0.2440)
Age	-0.0001	0.0037	0.0068
	(0.0011)	(0.0021)	(0.0047)
Ideological Congruence	0.3105***	0.6521***	1.223***
	(0.0357)	(0.0893)	(0.1486)
Education	0.0014	-0.0363	-0.0257
	(0.0169)	(0.0228)	(0.0596)
Gender (Female dummy)	-0.0014	-0.0875	-0.0488
	(0.0348)	(0.0822)	(0.1168)
Income	-0.0067	-0.0500	-0.0760
	(0.0219)	(0.0423)	(0.0878)
Fixed-effects			
Article	Yes	Yes	Yes
Fit statistics			
Observations	1,005	1,005	1,005
R^2	0.14592	0.17707	0.16216
Within R^2	0.08924	0.10140	0.09238

Clustered (Article & Respondent) standard-errors in parentheses
*Signif. Codes: ***: 0.001, **: 0.01, *: 0.05*

Table S32: Results from OLS Regression Results Presented in Figure 2d (50-75 Percentage Quartile of News Quality). This table presents the average treatment effects for linear regression models testing the effect of SOTEN. All effects are estimated using ordinary least squares (OLS) with article fixed effects and standard errors clustered at the individual and article level.

Dependent Variables:	Categorical Scale (True = 1)	Ordinal Scale (4-Point)	Ordinal Scale (7-Point)
Model:	(1)	(2)	(3)
Variables			
Treatment (Search)	0.0120	-0.0769	-0.1911
	(0.0469)	(0.0731)	(0.1619)
Age	0.0005	0.0036	0.0068
	(0.0011)	(0.0018)	(0.0048)
Ideological Congruence	0.2971***	0.6596***	1.218***
	(0.0393)	(0.0916)	(0.1619)
Education	0.0080	-0.0250	-0.0038
	(0.0157)	(0.0272)	(0.0588)
Gender (Female dummy)	0.0077	-0.0696	-0.0476
	(0.0321)	(0.0761)	(0.1334)
Income	-0.0183	-0.0691	-0.1064
	(0.0216)	(0.0444)	(0.0888)
Fixed-effects			
Article	Yes	Yes	Yes
Fit statistics			
Observations	1,006	1,006	1,006
R^2	0.13481	0.17948	0.16428
Within R^2	0.07046	0.09590	0.08667

Clustered (Article & Respondent) standard-errors in parentheses
*Signif. Codes: ***: 0.001, **: 0.01, *: 0.05*

Table S33: Results from OLS Regression Results Presented in Figure 2d (75-100 Percentage Quartile of News Quality). This table presents the average treatment effects for linear regression models testing the effect of SOTEN. All effects are estimated using ordinary least squares (OLS) with article fixed effects and standard errors clustered at the individual and article level.

Dependent Variables:	Categorical Scale (True = 1)	Ordinal Scale (4-Point)	Ordinal Scale (7-Point)
Model:	(1)	(2)	(3)
Variables			
Treatment (Search)	0.0449	0.0126	-0.1390
	(0.0545)	(0.1175)	(0.2324)
Age	0.0006	0.0046*	0.0096
	(0.0013)	(0.0020)	(0.0053)
Ideological Congruence	0.3018***	0.6468***	1.237***
	(0.0374)	(0.0855)	(0.1539)
Education	0.0023	-0.0426	-0.0378
	(0.0159)	(0.0267)	(0.0539)
Gender (Female dummy)	-0.0075	-0.0727	-0.0759
	(0.0284)	(0.0713)	(0.1141)
Income	-0.0117	-0.0369	-0.0334
	(0.0191)	(0.0389)	(0.0730)
Fixed-effects			
Article	Yes	Yes	Yes
Fit statistics			
Observations	1,008	1,008	1,008
R^2	0.12842	0.16508	0.15710
Within R^2	0.07246	0.09146	0.08932

Clustered (Article & Respondent) standard-errors in parentheses
*Signif. Codes: ***: 0.001, **: 0.01, *: 0.05*

Figure 3a

Table S34: Results from OLS Regression Results Presented in Figure 3a. This table presents effects for a linear regression model. All effects are estimated using ordinary least squares (OLS) with article fixed effects and standard errors clustered at the individual and article level.

Dependent Variable:	Returns Unreliable News Source
Model:	(1)
Variables	
Age	0.0027
	(0.0018)
Gender (Female dummy)	0.0172
	(0.0538)
Education	0.0044
	(0.0194)
Income	-0.0145
	(0.0267)
Ideological Congruence	0.0812
	(0.0443)
Digital Literacy Score	-0.0043
	(0.0028)
Fixed-effects	
Article	Yes
Fit statistics	
Observations	501
R^2	0.16051
Within R^2	0.01766

Clustered (Article & Respondent) standard-errors in parentheses
*Signif. Codes: ***: 0.001, **: 0.01, *: 0.05*

Figure 3c

Table S35: Predicted Use of Headline or URL as a Search Query when Searching Online about Misinformation. All effects are estimated using ordinary least squares (OLS) with article fixed effects and standard errors clustered at the individual and article level.

Dependent Variable:	Headline_Link
Model:	(1)
Variables	
Age	5.06×10^{-5}
	(0.0014)
Gender (Female dummy)	0.0117
	(0.0375)
Education	0.0138
	(0.0099)
Income	0.0033
	(0.0135)
Ideological Congruence	0.0307
	(0.0293)
Digital Literacy	-0.0044*
	(0.0016)
Fixed-effects	
Article	Yes
Fit statistics	
Observations	930
R^2	0.05668
Within R^2	0.01775

Clustered (Article & Respondent) standard-errors in parentheses
*Signif. Codes: ***: 0.001, **: 0.01, *: 0.05*

Figure 4a

Table S36: Results from OLS Regression Results Presented in Figure 4a (True Articles - Study 1). This table presents the average treatment effects for linear regression models testing the effect of SOTEN. All effects are estimated using ordinary least squares (OLS) with article fixed effects and standard errors clustered at the individual and article level.

Dependent Variable:	Categorical (Rated as True)
Model:	(1)
Variables	
Treatment (Search)	0.0717***
	(0.0166)
Age	-0.0004
	(0.0005)
Ideological Congruence	0.1862***
	(0.0311)
Education	0.0191**
	(0.0065)
Gender (Female dummy)	-0.0334*
	(0.0157)
Income	-0.0025
	(0.0084)
Fixed-effects	
Article	Yes
Fit statistics	
Observations	6,269
R^2	0.13836
Within R^2	0.02723

Clustered (Article & Respondent) standard-errors in parentheses
*Signif. Codes: ***: 0.001, **: 0.01, *: 0.05*

Table S37: Results from OLS Regression Results Presented in Figure 4a (False/Misleading Articles - Study 1). This table presents the average treatment effects for linear regression models testing the effect of SOTEN. All effects are estimated using ordinary least squares (OLS) with article fixed effects and standard errors clustered at the individual and article level.

Dependent Variable:	Categorical (Rated as True)
Model:	(1)
Variables	
Treatment (Search)	0.0568*
	(0.0243)
Age	-0.0029**
	(0.0007)
Ideological Congruence	0.1737**
	(0.0425)
Education	-0.0066
	(0.0118)
Gender (Female dummy)	-0.0329
	(0.0226)
Income	-0.0021
	(0.0099)
Fixed-effects	
Article	Yes
Fit statistics	
Observations	2,275
R^2	0.05869
Within R^2	0.03110

Clustered (Article & Respondent) standard-errors in parentheses
*Signif. Codes: ***: 0.001, **: 0.01, *: 0.05*

Table S38: Results from OLS Regression Results Presented in Figure 4a (True Articles - Study 2). This table presents the average treatment effects for linear regression models testing the effect of SOTEN. All effects are estimated using ordinary least squares (OLS) with article fixed effects and standard errors clustered at the individual and article level.

Dependent Variable:	Categorical (Rated as True)
Model:	(1)
Variables	
Treatment (Search)	0.0212
	(0.0121)
Age	-0.0001
	(0.0004)
Ideological Congruence	0.1683***
	(0.0332)
Education	0.0142
	(0.0078)
Gender (Female dummy)	-0.0152
	(0.0123)
Income	-0.0079
	(0.0088)
Fixed-effects	
Article	Yes
Fit statistics	
Observations	6,046
R^2	0.18543
Within R^2	0.01476

Clustered (Article & Respondent) standard-errors in parentheses
*Signif. Codes: ***: 0.001, **: 0.01, *: 0.05*

Table S39: Results from OLS Regression Results Presented in Figure 4a (False/Misleading Articles - Study 2). This table presents the average treatment effects for linear regression models testing the effect of SOTEN. All effects are estimated using ordinary least squares (OLS) with article fixed effects and standard errors clustered at the individual and article level.

Dependent Variable:	Categorical (Rated as True)
Model:	(1)
Variables	
Treatment (Search)	0.0713***
	(0.0159)
Age	-0.0021*
	(0.0008)
Ideological Congruence	0.1790***
	(0.0386)
Education	-0.0138
	(0.0126)
Gender (Female dummy)	-0.0238
	(0.0322)
Income	0.0032
	(0.0135)
Fixed-effects	
Article	Yes
Fit statistics	
Observations	2,020
R^2	0.11677
Within R^2	0.03413

Clustered (Article & Respondent) standard-errors in parentheses
*Signif. Codes: ***: 0.001, **: 0.01, *: 0.05*

Table S40: Results from OLS Regression Results Presented in Figure 4a (True Articles - Study 3). This table presents the average treatment effects for linear regression models testing the effect of SOTEN. All effects are estimated using ordinary least squares (OLS) with article fixed effects and standard errors clustered at the individual and article level.

Dependent Variable:	Categorical (Rated as True)
Model:	(1)
Variables	
Treatment (Search)	0.0467***
	(0.0117)
Age	-0.0007
	(0.0005)
Ideological Congruence	0.2285***
	(0.0286)
Education	0.0131
	(0.0067)
Gender (Female dummy)	-0.0022
	(0.0136)
Income	-0.0061
	(0.0082)
Fixed-effects	
Article	Yes
Fit statistics	
Observations	5,908
R^2	0.15842
Within R^2	0.02567

Clustered (Article & Respondent) standard-errors in parentheses
*Signif. Codes: ***: 0.001, **: 0.01, *: 0.05*

Table S41: Results from OLS Regression Results Presented in Figure 4a (False/Misleading Articles - Study 3). This table presents the average treatment effects for linear regression models testing the effect of SOTEN. All effects are estimated using ordinary least squares (OLS) with article fixed effects and standard errors clustered at the individual and article level.

Dependent Variable:	Categorical (Rated as True)
Model:	(1)
Variables	
Treatment (Search)	0.0662**
	(0.0195)
Age	-0.0015
	(0.0008)
Ideological Congruence	0.1912***
	(0.0369)
Education	0.0089
	(0.0097)
Gender (Female dummy)	-0.0632*
	(0.0245)
Income	0.0111
	(0.0143)
Fixed-effects	
Article	Yes
Fit statistics	
Observations	1,964
R^2	0.10520
Within R^2	0.04113

Clustered (Article & Respondent) standard-errors in parentheses
*Signif. Codes: ***: 0.001, **: 0.01, *: 0.05*

Table S42: Results from OLS Regression Results Presented in Figure 4a (True Articles - Study 4). This table presents the average treatment effects for linear regression models testing the effect of SOTEN. All effects are estimated using ordinary least squares (OLS) with article fixed effects and standard errors clustered at the individual and article level.

Dependent Variable:	Categorical (Rated as True)
Model:	(1)
Variables	
Treatment (Search)	0.0296
	(0.0207)
Age	-0.0021*
	(0.0009)
Ideological Congruence	0.2202**
	(0.0666)
Education	0.0139
	(0.0118)
Gender (Female dummy)	-0.0380
	(0.0343)
Income	0.0172
	(0.0175)
Fixed-effects	
Article	Yes
Fit statistics	
Observations	1,420
R^2	0.07682
Within R^2	0.02691

Clustered (Article & Respondent) standard-errors in parentheses
*Signif. Codes: ***: 0.001, **: 0.01, *: 0.05*

Table S43: Results from OLS Regression Results Presented in Figure 4a (False/Misleading Articles - Study 4). This table presents the average treatment effects for linear regression models testing the effect of SOTEN. All effects are estimated using ordinary least squares (OLS) with article fixed effects and standard errors clustered at the individual and article level.

Dependent Variable:	Categorical (Rated as True)
Model:	(1)
Variables	
Treatment (Search)	0.0674*
	(0.0301)
Age	-0.0039*
	(0.0013)
Ideological Congruence	0.1675*
	(0.0554)
Education	0.0344
	(0.0164)
Gender (Female dummy)	-0.0281
	(0.0534)
Income	0.0039
	(0.0240)
Fixed-effects	
Article	Yes
Fit statistics	
Observations	772
R^2	0.09822
Within R^2	0.04502

Clustered (Article & Respondent) standard-errors in parentheses
*Signif. Codes: ***: 0.001, **: 0.01, *: 0.05*

Table S44: Results from OLS Regression Results Presented in Figure 4a (True Articles - Study 5). This table presents the average treatment effects for linear regression models testing the effect of SOTEN. All effects are estimated using ordinary least squares (OLS) with article fixed effects and standard errors clustered at the individual and article level.

Dependent Variable:	Categorical (Rated as True)
Model:	(1)
Variables	
Treatment (Search)	0.1515***
	(0.0204)
Age	-0.0010
	(0.0006)
Ideological Congruence	0.1605***
	(0.0240)
Education	-0.0059
	(0.0081)
Gender (Female dummy)	0.0345*
	(0.0153)
Income	-0.0015
	(0.0082)
Fixed-effects	
Article	Yes
Fit statistics	
Observations	3,141
R^2	0.14659
Within R^2	0.05268

Clustered (Article & Respondent) standard-errors in parentheses
*Signif. Codes: ***: 0.001, **: 0.01, *: 0.05*

Table S45: Results from OLS Regression Results Presented in Figure 4a (False/Misleading Articles - Study 5). This table presents the average treatment effects for linear regression models testing the effect of SOTEN. All effects are estimated using ordinary least squares (OLS) with article fixed effects and standard errors clustered at the individual and article level.

Dependent Variable:	Categorical (Rated as True)
Model:	(1)
Variables	
Treatment (Search)	0.1068*
	(0.0388)
Age	0.0006
	(0.0009)
Ideological Congruence	0.2799***
	(0.0388)
Education	-0.0059
	(0.0144)
Gender (Female dummy)	0.0068
	(0.0279)
Income	-0.0058
	(0.0162)
Fixed-effects	
Article	Yes
Fit statistics	
Observations	1,485
R^2	0.12286
Within R^2	0.07909

Clustered (Article & Respondent) standard-errors in parentheses
*Signif. Codes: ***: 0.001, **: 0.01, *: 0.05*

Figure 4b

Table S46: Results from OLS Regression Results Presented in Figure 4b (True Low Quality Articles - Study 1). This table presents the average treatment effects for linear regression models testing the effect of SOTEN. All effects are estimated using ordinary least squares (OLS) with article fixed effects and standard errors clustered at the individual and article level.

Dependent Variable:	Categorical (Rated as True)
Model:	(1)
Variables	
Treatment (Search)	0.1052***
	(0.0258)
Age	-0.0005
	(0.0007)
Ideological Congruence	0.1674**
	(0.0417)
Education	0.0248*
	(0.0102)
Gender (Female dummy)	-0.0449
	(0.0230)
Income	-0.0297*
	(0.0113)
Fixed-effects	
Article	Yes
Fit statistics	
Observations	2,782
R^2	0.12549
Within R^2	0.04057

Clustered (Article & Respondent) standard-errors in parentheses
*Signif. Codes: ***: 0.001, **: 0.01, *: 0.05*

Table S47: Results from OLS Regression Results Presented in Figure 4b (True Mainstream Articles - Study 1). This table presents the average treatment effects for linear regression models testing the effect of SOTEN. All effects are estimated using ordinary least squares (OLS) with article fixed effects and standard errors clustered at the individual and article level.

Dependent Variable:	Categorical (Rated as True)
Model:	(1)
Variables	
Treatment (Search)	0.0453*
	(0.0173)
Age	-0.0003
	(0.0005)
Ideological Congruence	0.2245***
	(0.0306)
Education	0.0149
	(0.0074)
Gender (Female dummy)	-0.0225
	(0.0190)
Income	0.0191*
	(0.0081)
Fixed-effects	
Article	Yes
Fit statistics	
Observations	3,487
R^2	0.10122
Within R^2	0.02287

Clustered (Article & Respondent) standard-errors in parentheses
*Signif. Codes: ***: 0.001, **: 0.01, *: 0.05*

Table S48: Results from OLS Regression Results Presented in Figure 4b (False/Misleading Articles - Study 1). This table presents the average treatment effects for linear regression models testing the effect of SOTEN. All effects are estimated using ordinary least squares (OLS) with article fixed effects and standard errors clustered at the individual and article level.

Dependent Variable:	Categorical (Rated as True)
Model:	(1)
Variables	
Treatment (Search)	0.0568*
	(0.0243)
Age	-0.0029**
	(0.0007)
Ideological Congruence	0.1737**
	(0.0425)
Education	-0.0066
	(0.0118)
Gender (Female dummy)	-0.0329
	(0.0226)
Income	-0.0021
	(0.0099)
Fixed-effects	
Article	Yes
Fit statistics	
Observations	2,275
R^2	0.05869
Within R^2	0.03110

Clustered (Article & Respondent) standard-errors in parentheses
*Signif. Codes: ***: 0.001, **: 0.01, *: 0.05*

Table S49: Results from OLS Regression Results Presented in Figure 4b (True Low Quality Articles - Study 2). This table presents the average treatment effects for linear regression models testing the effect of SOTEN. All effects are estimated using ordinary least squares (OLS) with article fixed effects and standard errors clustered at the individual and article level.

Dependent Variable:	Categorical (Rated as True)
Model:	(1)
Variables	
Treatment (Search)	0.0809***
	(0.0185)
Age	0.0003
	(0.0007)
Ideological Congruence	0.1449**
	(0.0421)
Education	0.0138
	(0.0146)
Gender (Female dummy)	-0.0403*
	(0.0189)
Income	-0.0234
	(0.0117)
Fixed-effects	
Article	Yes
Fit statistics	
Observations	2,596
R^2	0.14979
Within R^2	0.02542

Clustered (Article & Respondent) standard-errors in parentheses
*Signif. Codes: ***: 0.001, **: 0.01, *: 0.05*

Table S50: Results from OLS Regression Results Presented in Figure 4b (True Mainstream Articles - Study 2). This table presents the average treatment effects for linear regression models testing the effect of SOTEN. All effects are estimated using ordinary least squares (OLS) with article fixed effects and standard errors clustered at the individual and article level.

Dependent Variable:	Categorical (Rated as True)
Model:	(1)
Variables	
Treatment (Search)	-0.0238
	(0.0128)
Age	-0.0005
	(0.0005)
Ideological Congruence	0.2253***
	(0.0482)
Education	0.0153
	(0.0085)
Gender (Female dummy)	0.0034
	(0.0160)
Income	0.0025
	(0.0126)
Fixed-effects	
Article	Yes
Fit statistics	
Observations	3,450
R^2	0.12520
Within R^2	0.01513

Clustered (Article & Respondent) standard-errors in parentheses
*Signif. Codes: ***: 0.001, **: 0.01, *: 0.05*

Table S51: Results from OLS Regression Results Presented in Figure 4b (False/Misleading Articles - Study 2). This table presents the average treatment effects for linear regression models testing the effect of SOTEN. All effects are estimated using ordinary least squares (OLS) with article fixed effects and standard errors clustered at the individual and article level.

Dependent Variable:	Categorical (Rated as True)
Model:	(1)
Variables	
Treatment (Search)	0.0713***
	(0.0159)
Age	-0.0021*
	(0.0008)
Ideological Congruence	0.1790***
	(0.0386)
Education	-0.0138
	(0.0126)
Gender (Female dummy)	-0.0238
	(0.0322)
Income	0.0032
	(0.0135)
Fixed-effects	
Article	Yes
Fit statistics	
Observations	2,020
R^2	0.11677
Within R^2	0.03413

Clustered (Article & Respondent) standard-errors in parentheses
*Signif. Codes: ***: 0.001, **: 0.01, *: 0.05*

Table S52: Results from OLS Regression Results Presented in Figure 4b (True Low Quality Articles - Study 3). This table presents the average treatment effects for linear regression models testing the effect of SOTEN. All effects are estimated using ordinary least squares (OLS) with article fixed effects and standard errors clustered at the individual and article level.

Dependent Variable:	Categorical (Rated as True)
Model:	(1)
Variables	
Treatment (Search)	0.1149***
	(0.0130)
Age	-7.28×10^{-6}
	(0.0008)
Ideological Congruence	0.2220***
	(0.0374)
Education	-0.0042
	(0.0108)
Gender (Female dummy)	-0.0203
	(0.0244)
Income	0.0102
	(0.0140)
Fixed-effects	
Article	Yes
Fit statistics	
Observations	2,490
R^2	0.14594
Within R^2	0.04598

Clustered (Article & Respondent) standard-errors in parentheses
*Signif. Codes: ***: 0.001, **: 0.01, *: 0.05*

Table S53: Results from OLS Regression Results Presented in Figure 4b (True Mainstream Articles - Study 3). This table presents the average treatment effects for linear regression models testing the effect of SOTEN. All effects are estimated using ordinary least squares (OLS) with article fixed effects and standard errors clustered at the individual and article level.

Dependent Variable:	Categorical (Rated as True)
Model:	(1)
Variables	
Treatment (Search)	-0.0029
	(0.0145)
Age	-0.0011
	(0.0006)
Ideological Congruence	0.2494***
	(0.0397)
Education	0.0254**
	(0.0081)
Gender (Female dummy)	0.0131
	(0.0150)
Income	-0.0177
	(0.0095)
Fixed-effects	
Article	Yes
Fit statistics	
Observations	3,418
R^2	0.10218
Within R^2	0.01942

Clustered (Article & Respondent) standard-errors in parentheses
*Signif. Codes: ***: 0.001, **: 0.01, *: 0.05*

Table S54: Results from OLS Regression Results Presented in Figure 4b (False/Misleading Articles - Study 3). This table presents the average treatment effects for linear regression models testing the effect of SOTEN. All effects are estimated using ordinary least squares (OLS) with article fixed effects and standard errors clustered at the individual and article level.

Dependent Variable:	Categorical (Rated as True)
Model:	(1)
Variables	
Treatment (Search)	0.0662**
	(0.0195)
Age	-0.0015
	(0.0008)
Ideological Congruence	0.1912***
	(0.0369)
Education	0.0089
	(0.0097)
Gender (Female dummy)	-0.0632*
	(0.0245)
Income	0.0111
	(0.0143)
Fixed-effects	
Article	Yes
Fit statistics	
Observations	1,964
R^2	0.10520
Within R^2	0.04113

Clustered (Article & Respondent) standard-errors in parentheses
*Signif. Codes: ***: 0.001, **: 0.01, *: 0.05*

Table S55: Results from OLS Regression Results Presented in Figure 4b (True Low Quality Articles - Study 4). This table presents the average treatment effects for linear regression models testing the effect of SOTEN. All effects are estimated using ordinary least squares (OLS) with article fixed effects and standard errors clustered at the individual and article level.

Dependent Variable:	Categorical (Rated as True)
Model:	(1)
Variables	
Treatment (Search)	0.0853*
	(0.0357)
Age	-0.0024
	(0.0015)
Ideological Congruence	0.2736**
	(0.0732)
Education	0.0285
	(0.0238)
Gender (Female dummy)	-0.0758
	(0.0542)
Income	-0.0124
	(0.0309)
Fixed-effects	
Article	Yes
Fit statistics	
Observations	516
R^2	0.10059
Within R^2	0.06149

Clustered (Article & Respondent) standard-errors in parentheses
*Signif. Codes: ***: 0.001, **: 0.01, *: 0.05*

Table S56: Results from OLS Regression Results Presented in Figure 4b (True Mainstream Articles - Study 4). This table presents the average treatment effects for linear regression models testing the effect of SOTEN. All effects are estimated using ordinary least squares (OLS) with article fixed effects and standard errors clustered at the individual and article level.

Dependent Variable:	Categorical (Rated as True)
Model:	(1)
Variables	
Treatment (Search)	-0.0022
	(0.0225)
Age	-0.0021
	(0.0011)
Ideological Congruence	0.0897
	(0.0612)
Education	0.0066
	(0.0140)
Gender (Female dummy)	-0.0166
	(0.0459)
Income	0.0328
	(0.0209)
Fixed-effects	
Article	Yes
Fit statistics	
Observations	904
R^2	0.04582
Within R^2	0.01510

Clustered (Article & Respondent) standard-errors in parentheses
*Signif. Codes: ***: 0.001, **: 0.01, *: 0.05*

Table S57: Results from OLS Regression Results Presented in Figure 4b (False/Misleading Articles - Study 4). This table presents the average treatment effects for linear regression models testing the effect of SOTEN. All effects are estimated using ordinary least squares (OLS) with article fixed effects and standard errors clustered at the individual and article level.

Dependent Variable:	Categorical (Rated as True)
Model:	(1)
Variables	
Treatment (Search)	0.0674*
	(0.0301)
Age	-0.0039*
	(0.0013)
Ideological Congruence	0.1675*
	(0.0554)
Education	0.0344
	(0.0164)
Gender (Female dummy)	-0.0281
	(0.0534)
Income	0.0039
	(0.0240)
Fixed-effects	
Article	Yes
Fit statistics	
Observations	772
R^2	0.09822
Within R^2	0.04502

Clustered (Article & Respondent) standard-errors in parentheses
*Signif. Codes: ***: 0.001, **: 0.01, *: 0.05*

Table S58: Results from OLS Regression Results Presented in Figure 4b (True Low Quality Articles - Study 5). This table presents the average treatment effects for linear regression models testing the effect of SOTEN. All effects are estimated using ordinary least squares (OLS) with article fixed effects and standard errors clustered at the individual and article level.

Dependent Variable:	Categorical (Rated as True)
Model:	(1)
Variables	
Treatment (Search)	0.2295***
	(0.0227)
Age	-0.0001
	(0.0012)
Ideological Congruence	0.1636***
	(0.0282)
Education	-0.0011
	(0.0133)
Gender (Female dummy)	0.0496
	(0.0263)
Income	0.0132
	(0.0125)
Fixed-effects	
Article	Yes
Fit statistics	
Observations	1,350
R^2	0.11991
Within R^2	0.08939

Clustered (Article & Respondent) standard-errors in parentheses
*Signif. Codes: ***: 0.001, **: 0.01, *: 0.05*

Table S59: Results from OLS Regression Results Presented in Figure 4b (True Mainstream Articles - Study 5). This table presents the average treatment effects for linear regression models testing the effect of SOTEN. All effects are estimated using ordinary least squares (OLS) with article fixed effects and standard errors clustered at the individual and article level.

Dependent Variable:	Categorical (Rated as True)
Model:	(1)
Variables	
Treatment (Search)	0.0905***
	(0.0231)
Age	-0.0017*
	(0.0006)
Ideological Congruence	0.1433**
	(0.0415)
Education	-0.0112
	(0.0100)
Gender (Female dummy)	0.0223
	(0.0179)
Income	-0.0149
	(0.0097)
Fixed-effects	
Article	Yes
Fit statistics	
Observations	1,791
R^2	0.15359
Within R^2	0.02806

Clustered (Article & Respondent) standard-errors in parentheses
*Signif. Codes: ***: 0.001, **: 0.01, *: 0.05*

Table S60: Results from OLS Regression Results Presented in Figure 4b (False/Misleading Articles - Study 5). This table presents the average treatment effects for linear regression models testing the effect of SOTEN. All effects are estimated using ordinary least squares (OLS) with article fixed effects and standard errors clustered at the individual and article level.

Dependent Variable:	Categorical (Rated as True)
Model:	(1)
Variables	
Treatment (Search)	0.1068*
	(0.0388)
Age	0.0006
	(0.0009)
Ideological Congruence	0.2799***
	(0.0388)
Education	-0.0059
	(0.0144)
Gender (Female dummy)	0.0068
	(0.0279)
Income	-0.0058
	(0.0162)
Fixed-effects	
Article	Yes
Fit statistics	
Observations	1,485
R^2	0.12286
Within R^2	0.07909

Clustered (Article & Respondent) standard-errors in parentheses
*Signif. Codes: ***: 0.001, **: 0.01, *: 0.05*

Figure 4c

Table S61: Results from OLS Regression Results Presented in Figure 4c (Between-Respondent Experiment - True Low-Quality Articles). This table presents the average treatment effects for linear regression models testing the effect of SOTEN. All effects are estimated using ordinary least squares (OLS) with article fixed effects and standard errors clustered at the individual and article level.

Dependent Variable:	Categorical (Rated as True)
Model:	(1)
Variables	
Treatment (Search)	0.1493***
	(0.0212)
Age	-0.0004
	(0.0006)
Ideological Congruence	0.1687***
	(0.0289)
Education	0.0170
	(0.0085)
Gender (Female dummy)	-0.0121
	(0.0186)
Income	-0.0114
	(0.0096)
Fixed-effects	
Article	Yes
Fit statistics	
Observations	4,132
R^2	0.14541
Within R^2	0.04830

Clustered (Article & Respondent) standard-errors in parentheses
*Signif. Codes: ***: 0.001, **: 0.01, *: 0.05*

Table S62: Results from OLS Regression Results Presented in Figure 4c (Within-Respondent Experiment - True Low-Quality Articles). This table presents the average treatment effects for linear regression models testing the effect of SOTEN. All effects are estimated using ordinary least squares (OLS) with article fixed effects and standard errors clustered at the individual and article level.

Dependent Variable:	Categorical (Rated as True)
Model:	(1)
Variables	
Treatment (Search)	0.0964***
	(0.0119)
Age	-0.0002
	(0.0006)
Ideological Congruence	0.1936***
	(0.0305)
Education	0.0058
	(0.0081)
Gender (Female dummy)	-0.0304*
	(0.0136)
Income	-0.0056
	(0.0089)
Fixed-effects	
Article	Yes
Fit statistics	
Observations	5,602
R^2	0.13110
Within R^2	0.03490

Clustered (Article & Respondent) standard-errors in parentheses
*Signif. Codes: ***: 0.001, **: 0.01, *: 0.05*

Table S63: Results from OLS Regression Results Presented in Figure 4c (Between-Respondent Experiment - True Mainstream Articles). This table presents the average treatment effects for linear regression models testing the effect of SOTEN. All effects are estimated using ordinary least squares (OLS) with article fixed effects and standard errors clustered at the individual and article level.

Dependent Variable:	Categorical (Rated as True)
Model:	(1)
Variables	
Treatment (Search)	0.0602***
	(0.0143)
Age	-0.0004
	(0.0005)
Ideological Congruence	0.2051***
	(0.0277)
Education	0.0079
	(0.0062)
Gender (Female dummy)	-0.0126
	(0.0136)
Income	0.0130
	(0.0066)
Fixed-effects	
Article	Yes
Fit statistics	
Observations	5,278
R^2	0.12869
Within R^2	0.01898

Clustered (Article & Respondent) standard-errors in parentheses
*Signif. Codes: ***: 0.001, **: 0.01, *: 0.05*

Table S64: Results from OLS Regression Results Presented in Figure 4c (Within-Respondent Experiment - True Mainstream Articles). This table presents the average treatment effects for linear regression models testing the effect of SOTEN. All effects are estimated using ordinary least squares (OLS) with article fixed effects and standard errors clustered at the individual and article level.

Dependent Variable:	Categorical (Rated as True)
Model:	(1)
Variables	
Treatment (Search)	-0.0121
	(0.0103)
Age	-0.0009*
	(0.0004)
Ideological Congruence	0.2270***
	(0.0363)
Education	0.0185***
	(0.0048)
Gender (Female dummy)	0.0055
	(0.0101)
Income	-0.0022
	(0.0079)
Fixed-effects	
Article	Yes
Fit statistics	
Observations	7,772
R^2	0.09534
Within R^2	0.01445

Clustered (Article & Respondent) standard-errors in parentheses
*Signif. Codes: ***: 0.001, **: 0.01, *: 0.05*

Table S65: Results from OLS Regression Results Presented in Figure 4c (Between-Respondent Experiment - False/Misleading Articles). This table presents the average treatment effects for linear regression models testing the effect of SOTEN. All effects are estimated using ordinary least squares (OLS) with article fixed effects and standard errors clustered at the individual and article level.

Dependent Variable:	Categorical (Rated as True)
Model:	(1)
Variables	
Treatment (Search)	0.0685^{***}
	(0.0126)
Age	-0.0024^{***}
	(0.0005)
Ideological Congruence	0.1836^{***}
	(0.0243)
Education	0.0031
	(0.0082)
Gender (Female dummy)	-0.0376
	(0.0190)
Income	0.0029
	(0.0096)
Fixed-effects	
Article	Yes
Fit statistics	
Observations	4,756
R^2	0.09062
Within R^2	0.03589

Clustered (Article & Respondent) standard-errors in parentheses
*Signif. Codes: ***: 0.001, **: 0.01, *: 0.05*

Table S66: Results from OLS Regression Results Presented in Figure 4c (Within-Respondent Experiment - False/Misleading Articles). This table presents the average treatment effects for linear regression models testing the effect of SOTEN. All effects are estimated using ordinary least squares (OLS) with article fixed effects and standard errors clustered at the individual and article level.

Dependent Variable:	Categorical (Rated as True)
Model:	(1)
Variables	
Treatment (Search)	0.0759**
	(0.0214)
Age	-0.0020**
	(0.0006)
Ideological Congruence	0.2270***
	(0.0313)
Education	-0.0054
	(0.0091)
Gender (Female dummy)	-0.0136
	(0.0181)
Income	-0.0014
	(0.0087)
Fixed-effects	
Article	Yes
Fit statistics	
Observations	3,760
R^2	0.09010
Within R^2	0.04544

Clustered (Article & Respondent) standard-errors in parentheses
*Signif. Codes: ***: 0.001, **: 0.01, *: 0.05*

C Descriptive Statistics for NewsGuard Ratings

A histogram of NewsGuard scores is listed below:

Figure S1: Histogram of NewsGuard Scores

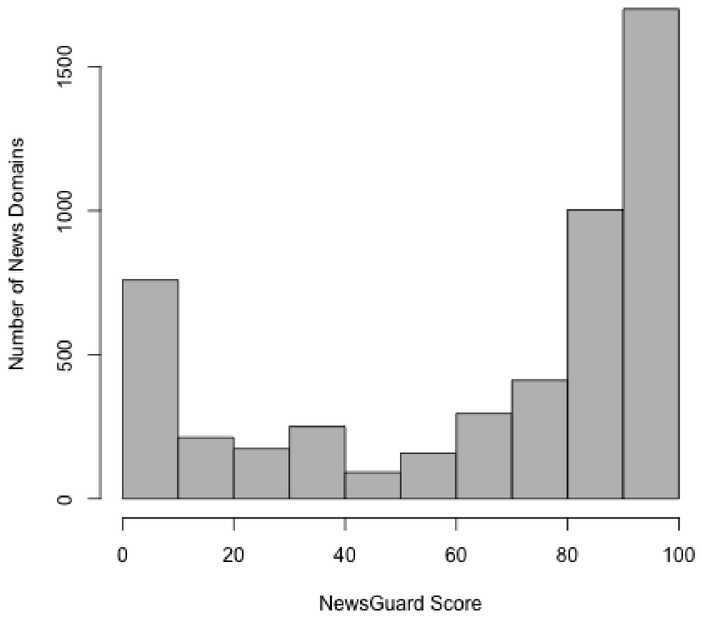

D Explanation of Sampling Technique for Respondents

Given that internet surveys using opt-in panels are the less accurate than probability sampling (MacInnis et al., 2018), we must be cautious when experimental results using non-probability sampling from Qualtrics or Mechanical Turk. In this paper, we only report results from analyses using non-probability sampling. Given that this is an opt-in survey, we expect the behavior of these respondents who self-selected in into the survey to differ from those drawn with known probability from a well-specified population. Therefore, it is possible and likely this convenience sample is different in unmeasured ways. We must be cautious when making experimental inferences using an opt-in non-probability samples from Qualtrics, but previous work has found that about 90% of effects identified using a gold-standard probability sample are similar to effects identified by an opt-in Qualtrics panel (Zack et al., 2019). The behaviors of those who opt-in to and join multiple panels to earn incentives may put much less effort into tasks at hand and are more likely to guess in order to save time and maximize their payment. To test if this would affect our main results, we ran a parallel survey and paid respondents additional payments for correct answers to our veracity question, but did not find significant differences in their responses. Therefore, we do not believe that a lack of effort explain the results we find. Recent work has also shown that experimental results from these non-probability samples are often comparable to those found in population samples (Mullinix et al., 2015). Given this previous work, the results we present are not likely to be different if we had used probability-sampling.

E Article Selection Process

E.1 Mainstream News Sites We Sourced From

Mainstream Liberal News Sites:

- Yahoo News
- The New York Times
- The Huffington Post
- NBC News
- Politico
- CNN
- The Washington Post
- The Guardian
- USA Today
- CBS News

Mainstream Conservative News Sites:

Note: Conservative news group contains only nine websites. The Drudge report did not have a Facebook page, and therefore could not be followed on CrowdTangle. Since there were only ten conservative leaning websites in the top 100 list, we used the only nine that had Facebook pages.

- Fox News
- The New York Post
- Real Clear Politics
- IJR
- The Washington Times
- CNBC
- The Wall Street Journal
- Newsmax
- Townhall

E.2 Low-Quality News Sites We Sourced From

Table S67: Low-Quality Conservative Sources

Number	Domain
1	dailywire.com
2	dailycaller.com
3	express.co.uk
4	redstatewatcher.com
5	thepoliticalinsider.com
6	thefederalistpapers.org
7	rightwingnews.com
8	madworldnews.com
9	yournewswire.com
10	uschronicle.com
11	louderwithcrowder.com
12	100percentfedup.com
13	angrypatriotmovement.com
14	ilovemyfreedom.org
15	clashdaily.com
16	joeforamerica.com
17	conservativedailypost.com
18	americasfreedomfighters.com
19	babylonbee.com
20	teaparty.org
21	judicialwatch.org
22	conservativepost.com
23	thegatewaypundit.com
24	infowars.com
25	eaglerising.com
26	en-volve.com
27	wnd.com
28	bb4sp.com
29	concealednation.org
30	theconservativetreehouse.com
31	dcclothesline.com
32	conservativefiringline.com
33	frontpagemag.com
34	endtimeheadlines.org
35	downtrend.com
36	nowtheendbegins.com
37	wearechange.org
38	neonnettle.com
39	powderedwigsociety.com
40	americanjournalreview.com
41	thehornnews.com
42	barenakedislam.com
43	rickwells.us

Table S68: Low-Quality Conservative Sources (Continued)

Number	Domain
44	ahtribune.com
45	ipatriot.com
46	afa.net
47	eutimes.net
48	thepeoplescube.com
49	stateofthenation2012.com
50	fellowshipoftheminds.com
51	trunews.com
52	freerepublic.com
53	mediamass.net
54	endoftheamericandream.com
55	2ndvote.com
56	iotwreport.com
57	puppetstringnews.com
58	dailyheadlines.net
59	thenationalpatriot.com
60	rogue-nation3.com
61	veteransfordonaldtrump.com

Table S69: Low-Quality Liberal Sources

Number	Domain
1	occupydemocrats.com
2	bipartisanreport.com
3	palmerreport.com
4	crooksandliars.com
5	democraticunderground.com
6	halfwaypost.com

Table S70: Low-Quality Unclear Sources

Number	Domain
1	ijr.com
2	anonhq.com
3	inquisitr.com
4	worldtruth.tv
5	collective-evolution.com
6	tribunist.com
7	naturalnews.com
8	worldnewsdailyreport.com
9	trueactivist.com
10	firstpost.com
11	zerohedge.com
12	disclose.tv
13	dailysnark.com
14	postcard.news
15	higherperspectives.com
16	dailypost.ng
17	davidwolfe.com
18	noticias-frescas.com
19	healthnutnews.com
20	beforeitsnews.com
21	truthuncensored.net
22	awarenessact.com
23	duffelblog.com
24	nation.com.pk
25	actualidadpanamericana.com
26	themindunleashed.com
27	huzlers.com
28	dennismichaellynch.com
29	rearfront.com
30	actualite.co
31	activistpost.com
32	newzmagazine.com
33	12minutos.com
34	dailyoccupation.com
35	newsrescue.com
36	the-postillon.com
37	burrardstreetjournal.com
38	empirenews.net
39	medicalkidnap.com
40	friendsofsyria.wordpress.com
41	realnewsrightnow.com
42	adobochronicles.com
43	anonews.co

Table S71: Low-Quality Unclear Sources (Continued)

Number	Domain
44	thenationalmarijuananews.com
45	en.mediamass.net
46	daily-sun.com
47	whatdoesitmean.com
48	therooster.com
49	thelastamericanvagabond.com
50	stillnessinthestorm.com
51	independentminute.com
52	newsbiscuit.com
53	attitude.co.uk
54	onlysimchas.com
55	dailyfeed.news
56	newsjustforyou1.blogspot.com
57	thebreakingnews.co
58	usanewstoday.com

E.3 Method for Determining Partisan Lean of News Sites

We determine the partisan lean of the low-quality domains by asking three independent coders to determine the partisan perspective of the website (conservative, liberal, and unclear). Coders were asked to use the headlines, the content of its articles, as well as the websites domain and about page to make this determination, and to classify websites that had a clear partisan affiliation based on this information accordingly. Websites were not classified as liberal or conservative unless at least 50% of their content appeared to have a partisan or political nature. If websites did not meet this threshold, they were classified as unclear. If the coders did not unanimously agree, a fourth coder was asked to evaluate the website, and the majority decision was used. There was over 75% level agreement among the coders, and we report a .705 Fleiss' Kappa. In total, six domains were placed in the liberal low-quality news stream, fifty domains were placed in the conservative low-quality news stream, and forty-three domains were placed in the unclear low-quality news stream. The prevalence of conservative and unclear low-quality news streams is in line with previous research that provides evidence for the asymmetric production of false/misleading news (Guess et al., 2020).

F Attention Checks

(1) Was it possible to access the article using the link provided?

(A) Yes (Correct)
(B) No (Incorrect)

(2) Is the full article blocked by some paywall or require a subscription (the website asks you to pay for article, or it requires a subscription to access the article and you do not have one)?

(A) Yes (Incorrect)
(B) No (Correct)

G Social Media Presence of Articles Selected in Studies 1–3

Table S72: Summary Statistics For Twitter Posts (within 48 hours of publication) Per News Stream

News Stream	Mean	Median	Max	Min
Conservative Low-Quality	90.065	14	1577	0
Liberal Low-Quality	222.97	52	2687	0
Unclear Low-Quality	1570.23	14	42696	0
Conservative Mainstream	999.48	197	5270	0
Liberal Mainstream	1270.19	1198	6702	0
All Low-Quality	627.75	21	42696	0
All Mainstream	1134.84	308	6702	0
All Articles	830.59	52	42696	0

Table S73: Summary Statistics For Facebook Shares Per News Stream

News Stream	Mean	Median	Max	Min
Conservative Low-Quality	587.52	116	5421	0
Liberal Low-Quality	917.39	265	9050	16
Unclear Low-Quality	9222.76	135	147127	0
Conservative Mainstream	26200.48	10643	190157	0
Liberal Mainstream	25808.00	16372	117365	195
All Low-Quality	3186.45	205	147127	0
All Mainstream	25990.71	13711	190157	0
All Articles	12308.15	1109	190157	0

H Additional Instructions to Search Online for Information

We now ask you to research this central claim by finding evidence supporting or contradicting it. Please use any internet based source you trust. By evidence, we mean a statement, photo, video, audio, or statistic relevant to the central claim. This evidence should be reported by a different source than the one whose content you are investigating. This evidence can either support the central claim or contradict it.

Guidance for the finding evidence for or against the central claim you've identified:

(1) By evidence, we mean an article, statement, photo, video, audio, or statistic relevant to the central claim. This evidence should be reported by some other source than the author of the article you are investigating. This evidence can either support the central claim or go against it.

(2) To evaluate the central claim, you will be asked to search for evidence about the claim and to provide some information about the evidence you found (the steps of this process are described in detail below).

(3) We ask that you use the highest quality pieces of evidence to evaluate the central claim in your search. If you cannot find evidence about the claim from a source that you trust, you should try to find the most relevant evidence about the claim you can find from any source, even one you don't trust. In the task, you will be asked to note whether you do not trust the evidence provided by the original source.

(4) DO NOT directly copy and paste the headline or title of the article you are evaluating into the search engine as your search terms.

(5) DO select key terms related to the central claim to enter into the search engine as your search terms.

(6) DO try using synonyms for different key terms if you're having trouble finding evidence from sources you trust.

(7) To locate relevant evidence, try to also include in your search terms the names of key people, locations, actions and events described in the article, when possible.

(8) For claims about current events, you should also include search terms related to when the event occurred or the link you are evaluating was published (e.g., the month and/or year) to help locate relevant evidence.

(9) DO NOT use extreme language. For example, if an article uses extreme language in the way it states its claim, searching using those same extreme terms may make you more likely to only encounter sources that agree with the article you are evaluating (vs more diverse perspectives or reporting on the claim). Therefore, when evaluating an article that uses extreme language, you should try searching using more neutral terms.

I Data Measurement – Studies 1–4

1. **Ideology of respondent:** We ask individuals to self-identify their ideology using the following question. The score they receive on the ideological scale is in parentheses next to the answer they give.

   Where would you place yourself on this scale?
   (A) Extremely Conservative (3)
   (B) Conservative (2)
   (C) Slightly Conservative (1)
   (D) Moderate (0)
   (E) Slightly Liberal (-1)
   (F) Liberal (-2)
   (G) Extremely Liberal (-3)
   (H) Haven't Thought Much About it (NA)

   Variable names used later in models: $Con\_Ideology\_Score_i$ is assigned the value that corresponds to the answer they choose.

2. **Digital Literacy:** Digital literacy is measured by asking for respondent's familiarity with the following terms: Phishing ; Hashtag ; Preference Setting ; Wiki ; PDF ; Malware ; RSS ; BCC (on email) ; Tablet ; Tagging. We ask them for their familiarity on a five point scale (1 representing no understanding and 5 representing full understanding). The digital literacy score for each respondent is the average of the scores across these categories.

   DL_Score_i is the digital literacy score a respondent receives.

3. **7-Point Ordinal Veracity Scale:** To determine how confident a respondent is of their evaluation we ask respondents the following question.

   Now that you have evaluated the article, we are interested in the strength of your opinion. Please rank the article on the following scale:
   (A) 1 - Definitely NOT TRUE
   (B) 2
   (C) 3
   (D) 4
   (B) 5
   (C) 6
   (D) 7 - Definitely TRUE

   The variable, $likert_i$, is assigned the value of the answer chosen by the respondent (1 through 7). The same question is assigned to a group of professional fact-checkers. We plan to compare the mean choice of the fact-checkers to the individual truth-likert value (μ_f) to determine the absolute difference between the average fact-checker value on the likert scale and the respondent's chosen value on the likert scale ($|likert_i - \mu_f|$):

4. **Asked to research information by survey:** If the respondent is apart of the treatment group and is asked to search for information to evaluate the article the variable $Treatment\_Search$ is assigned a one, otherwise it is assigned a zero.

5. **Education:** We ask individuals to self-identify their highest degree earned and attribute the following numeric value to each answer: No High School education (0) ; High School Education (1) ; Associates Degree (2) ; Bachelors Degree (3) ; Masters Degree (4) ; Doctorate Degree (5)

6. **Income:** We ask individuals to self-identify their income from last year and attribute the following numeric value to each answer: $0 - $50,000 (0) ; $50,000 - $100,000 (1) ; $100,000 - $150,000 (2) ; $150,000 plus (3)

7. **Gender (Female Dummy):** What is your gender? Male (0) ; Female (1) ; Other (0)

J Different Data Measurement – Study 5

1. **Digital Literacy:** Digital literacy is measured using the following two grid questions.

   The first grid question asks for respondent's familiarity with the following terms on a five point scale (1 representing no understanding and 5 representing full understanding):
   (1) Phishing
   (2) JPG
   (3) Cache
   (4) Malware
   (5) RSS
   (6) Hashtag

   The second grid question asks respondents' agreement with the following statements on a scale of -4 = Strongly Disagree to 4 = Strongly Agree:
   (1) I prefer to ask friends how to use any new technological gadget instead of trying to figure it out myself.
   (2) I feel like information technology is a part of my daily life.
   (3) Using information technology makes it easier to do my work.
   (4) I often have trouble finding things that I've saved on my computer.

   Note: The value for one and four are reverse coded.

   By summing all of the values we can create a digital literacy score that is assigned to the variable: DL_Score_i for each respondent. We take the inverse to determine if the effect size increased with lower levels of digital literacy.

   DL_Score_i is the digital literacy score a respondent receives.

2. **4-Point Ordinal Scale (Veracity Scale):** We asked respondents to evaluate the accuracy of a number of headlines on the following 4-point scale:
   (A) Not at all accurate (1) (B) Not very accurate (2) (C) Somewhat accurate (3) (D) Very accurate (4)

K Fact-Checker Agreement

Table S74: Inter-Rater Reliability Statistics for Fact-Checker Evaluations of Articles in Studies 1–5

Number of Articles Evaluated	Percentage of Articles with Unanimous Agreement	Fleiss Kappa
265	44.62	0.42

L Ideological Perspectives of Articles (Studies 1–4)

In Studies 1–4 we determine the partisan lean of the articles by asking four independent coders to determine the ideological perspective of the article (conservative, liberal, neutral and unclear). The partisan lean was determined by taking the modal evaluation of the coders. When there was no modal evaluation, or there was a tie, the evaluation of a graduate student was used as the tiebreaker. Coders were asked to use only the headline and content of the article to make their determination.In Study 5 we just asked our fact-checkers to rate them by their ideological perspective and took the modal classification. The following guidance was given to raters for selecting the partisan perspective:

Articles that are clearly written from a partisan perspective should be classified according to whichever direction that is, even if the article is not completely supportive of the political party that shares that ideology. Articles that are clearly advocating for one side of the political spectrum should be classified as leaning that way. Importantly, just because partisans may feel differently about an article, does not mean the article does not have a neutral perspective. For example, "Trump Impeached" may induce very different reactions among liberals and conservatives, but the article could still be neutral so long as it reports on this event objectively. Conversely, "Trump is a Crook" likely has a liberal perspective. Importantly, neutral articles are those where the perspective is balanced and appears to show no bias. Unclear articles are those where the perspective does not appear to be any of the three above or you are unable to make a clear determination.

Table S75: Inter-Rater Reliability Statistics for Ideological Perspective Ratings of Articles in Studies 1–5

Number of Articles	Unanimous Agreement	Fleiss Kappa
265.00	53.21%	0.60

M Figures Only Using Robust Modal Classification

Figure S2: The effect of searching online on belief in misinformation across Study 1 through 4. (Robust mode only) Panels a and b present the average treatment effect of SOTEN and 95 percent confidence intervals during Studies 1 (N = 1,236 total evaluations), 2 (N = 1,108), 3 (N = 1,078), and 4 (N = 556). All effects are estimated using ordinary least squares (OLS) with article fixed effects and standard errors clustered at the individual and article level. Panel a presents the effect of SOTEN on rating misinformation as true for Study 1 (P = 0.365), 2 (P=0.0277), 3 (P=0.252), and 4 (P=0.0653). Panel b presents the effect of SOTEN on a 7-point ordinal scale of veracity for Study 1 (P = 0.184), 2 (P=0.022), 3 (P=0.0154), and 4 (P=0.0216).

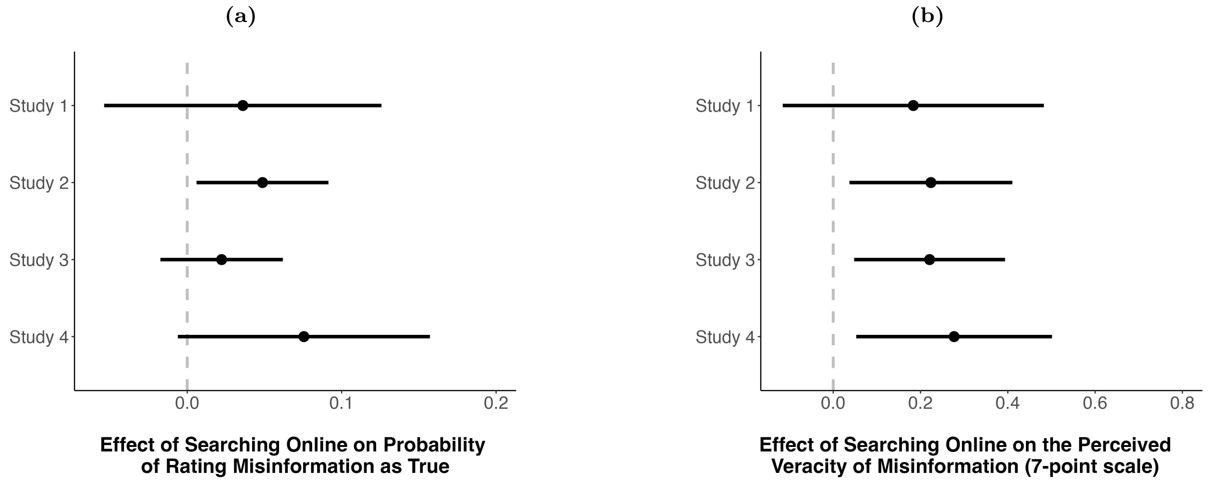

Figure S3: How do Google Search Results affect belief in misinformation? (Study 5, Robust mode only). Panel a presents the proportion of individuals who, when searching online about an article, are exposed to different levels of unreliable news sites in Google search results. We present these proportions for those searching about true articles and those searching about false/misleading articles. Panel b presents the average treatment effect of searching online on rating a false/misleading article as true and 95 percent confidence intervals during Study 5 (N = 973) as a unit of the standard deviation of the dependent variable. Searching online increased the probability a respondent rated a false or misleading article as true and increased the average score, but not on on a 4-point ordinal scale or a 7-point ordinal scale. Subsetting the treatment group by the quality of news returned in their search engine results, Panel c and d present these same marginal effects and 95 percent confidence intervals. Panel c shows that the probability an individual believes misinformation is higher than the control group among respondents who are exposed to at least one unreliable news site (N = 602) using the categorical measure and 4-point ordinal scale, but not the 7-point ordinal scale. Also, the probability an individual believes misinformation is not different than the control group among respondents who are exposed to only very reliable news (N = 533) using the categorical measure, the 4-point ordinal scale, and the the 7-point ordinal scale. Panel d shows that the probability an individual believes misinformation is higher than the control group among respondents who are exposed to the lowest quartile of news quality (N = 595) using the categorical measure, but not the 4-point ordinal scale or the 7-point ordinal scale. Also, the probability an individual believes misinformation is higher than the control group among respondents who are exposed to the second lowest quartile of news quality (N = 569) using the categorical measure and the 4-point ordinal scale, but not the 7-point ordinal scale. The probability an individual believes misinformation is not different than the control group among respondents who are exposed to the second highest quartile of news quality (N = 577) using the categorical measure, the 4-point ordinal scale, and the 7-point ordinal scale. The probability an individual believes misinformation is not different than the control group among respondents who are exposed to the highest quartile of news quality (N = 581) using the categorical measure, the 4-point ordinal scale, and the 7-point ordinal scale. All effects are estimated using ordinary least squares (OLS) with article fixed effects and standard errors clustered at the individual and article level.

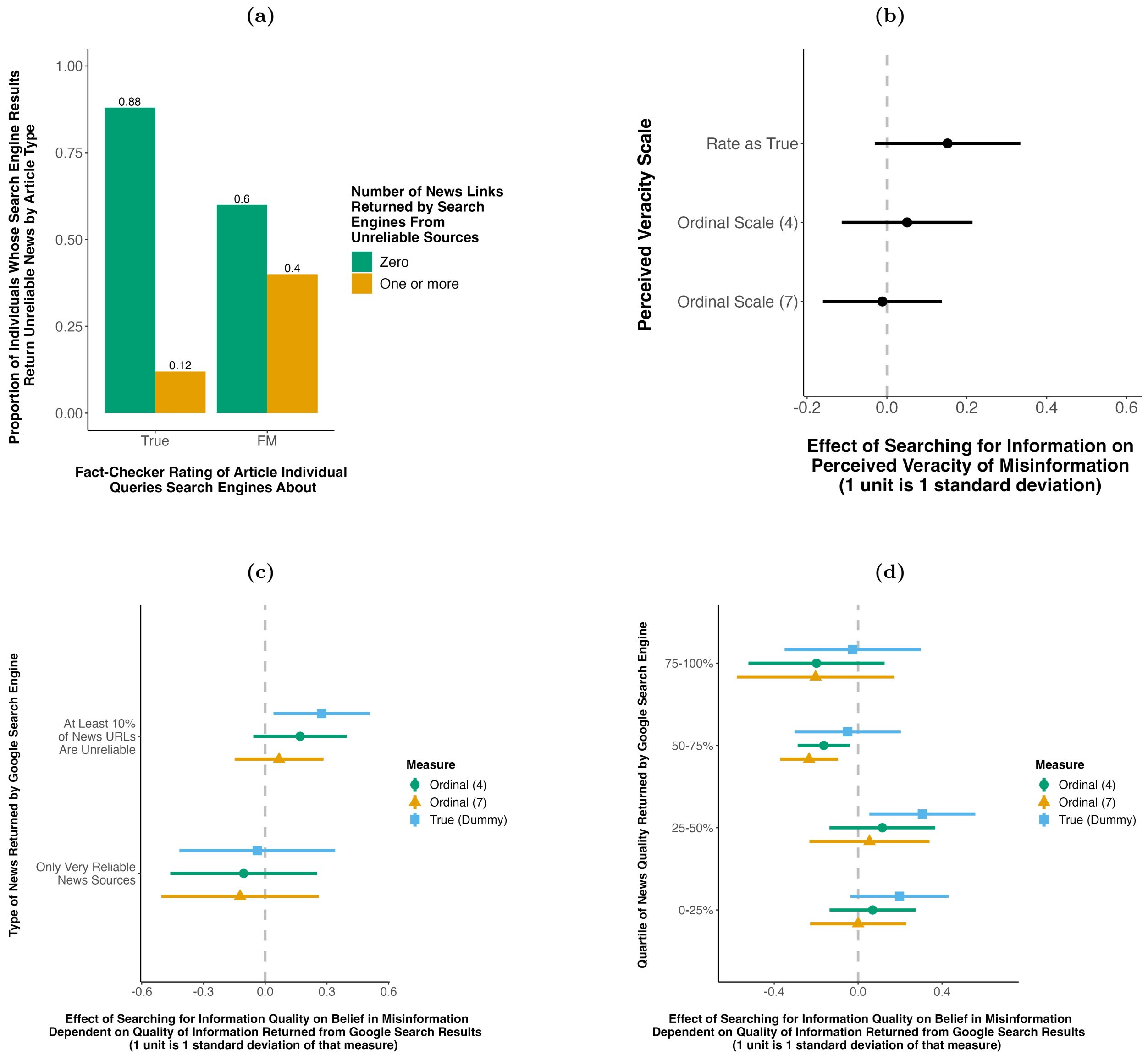

Figure S4: Who is exposed to unreliable news sites when evaluating misinformation online? (Study 5, Robust mode only). Panel a presents the effect of demographic variables on the probability of exposure to unreliable news sources when searching online about false/misleading news articles and 95 percent confidence intervals during Study 5 (N = 326). Panel b presents the proportion of online searches individuals engage in (N = 689) when searching online about a false/misleading article that return different levels of unreliable news sites by the Google search engine. We present these proportions for those who use the headline of the article or the link of the article and those who use another query. Panel c presents the effect of demographic variables on the probability of using the headline/lede or unique URL when searching online about false/misleading news articles and 95 percent confidence intervals during Study 5 (N = 689). All effects are estimated using ordinary least squares (OLS) with article fixed effects and standard errors clustered at the individual and article level.

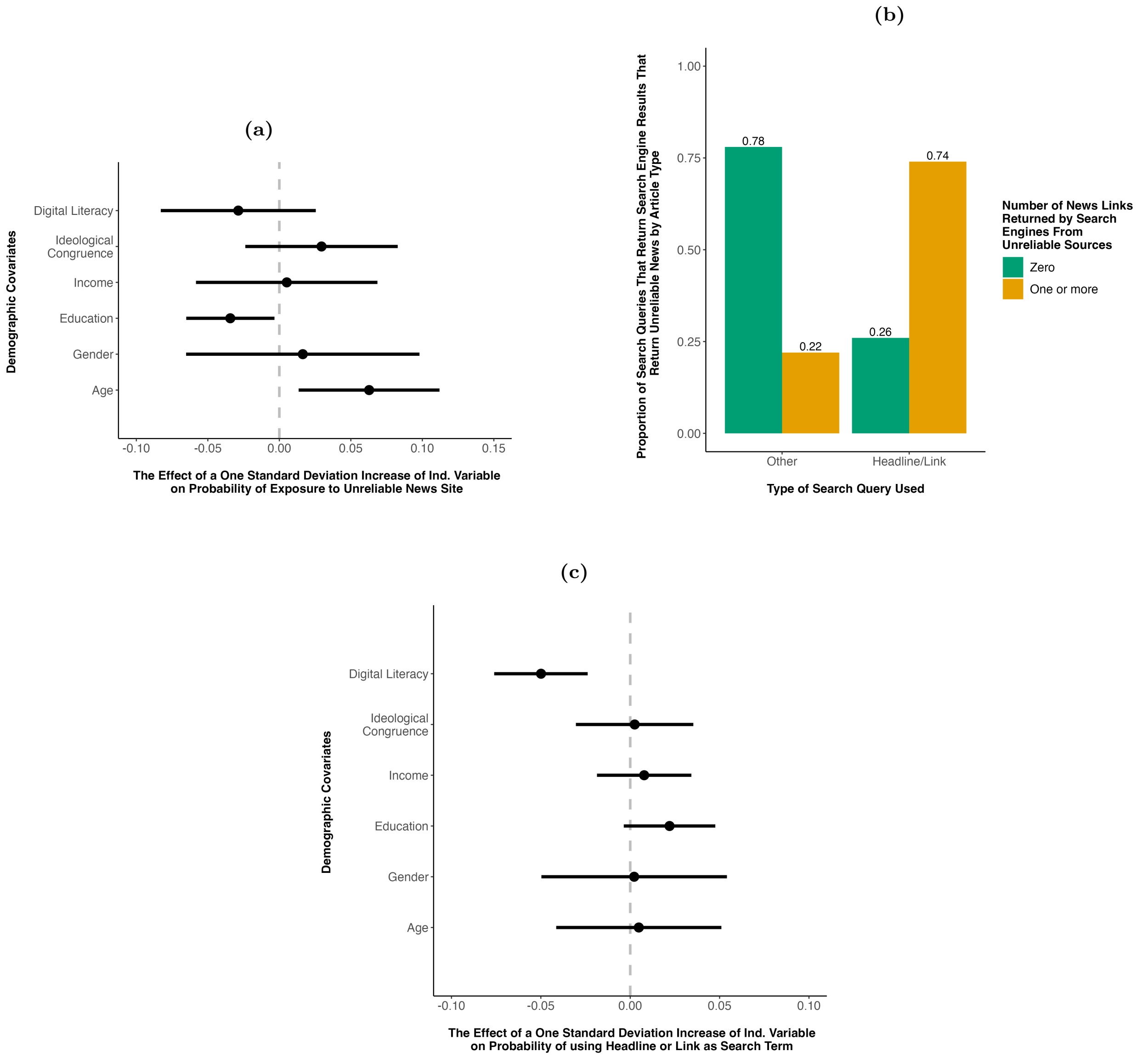

Figure S5: The effect of searching online to evaluate news on belief in false/misleading and true news (Robust mode only). Panel a presents the effect of rating true news as true and false/misleading news as true and 95 percent confidence intervals during Studies 1 (N = 4,369, N = 1,236), 2 (N = 4,728, N = 1,108), 3 (N = 4,646, N = 1,078), 4 (N = 1,208, N = 556), and 5 (N = 2,643, N = 973). Panel b the effect of rating true news as true from low-quality sources, true news as true from mainstream sources, and false/misleading news as true and 95 percent confidence intervals during Studies 1 (N = 1,047, N = 3,302, N = 1,236), 2 (N = 1,448, N = 3,280, N = 1,108), 3 (N = 1,347, N = 3,272, N = 1,078), 4 (N = 366, N = 842, N = 556), and 5 (N = 852, N = 1,791, N = 973). Panel c presents the effect of rating true news as true from low-quality sources, true news as true from mainstream sources, and false/misleading news as true and 95 percent confidence intervals for between-respondent experiments (Studies 1 and 5) (N = 1,899, N = 5,113, N = 2,209) and within-respondent experiments (Studies 2-4) (N = 3,188, N = 7,394, N = 2,742).

(a)

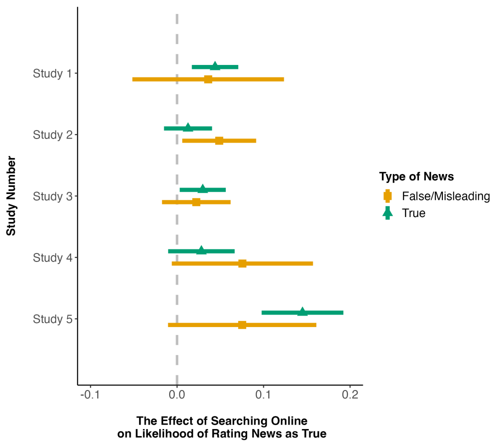

(b)

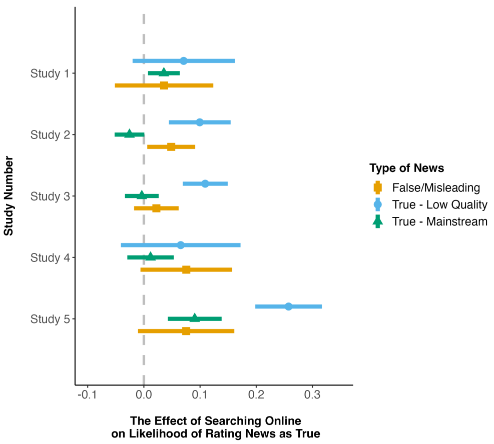

(c)

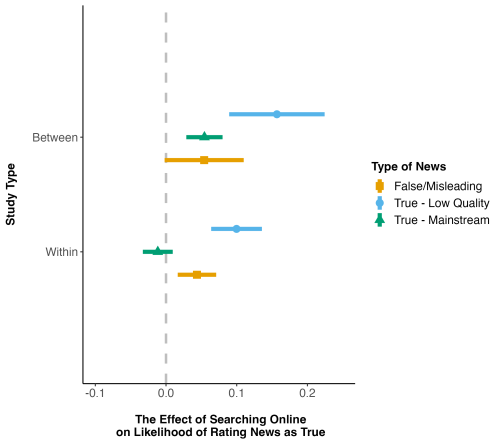

N Number of Unique Respondents and Evaluations in Studies 1-5

Table S76: Number of Unique Respondents and Evaluations in Study 1 by Article Type and Assigned Group

Article Type	Control or Treatment Group	Number of Unique Articles Evaluated	Number of Unique Respondents	Number of Evaluations
False/Misleading news	13	Control	876	1,145
False/Misleading news	13	Treatment	872	1,130
True news from a Low-Quality Source	16	Control	1,016	1,410
True news from a Low-Quality Source	16	Treatment	995	1,372
True news from a Mainstream Source	20	Control	1,312	1,735
True news from a Mainstream Source	20	Treatment	1,300	1,752

Table S77: Number of Unique Respondents and Evaluations in Study 2 by Article Type and Assigned Group

Article Type	Number of Unique Articles Evaluated	Number of Evaluations
False/Misleading news	33	1,010
True news from a Low-Quality Source	41	1,298
True news from a Mainstream Source	56	1,725

Table S78: Number of Unique Respondents and Evaluations in Study 3 by Article Type and Assigned Group

Article Type	Number of Unique Articles Evaluated	Number of Evaluations
False/Misleading news	33	982
True news from a Low-Quality Source	41	1,245
True news from a Mainstream Source	56	1,709

Table S79: Number of Unique Respondents and Evaluations in Study 4 by Article Type and Assigned Group

Article Type	Number of Unique Articles Evaluated	Number of Evaluations
False/Misleading news	13	386
True news from a Low-Quality Source	9	258
True news from a Mainstream Source	16	452

Table S80: Number of Unique Respondents and Evaluations in Study 5 by Article Type and Assigned Group

Article Type	Control or Treatment Group	Number of Unique Articles Evaluated	Number of Unique Respondents	Number of Evaluations
False/Misleading news	17	Control	621	877
False/Misleading news	17	Treatment	451	608
True news from a Low-Quality Source	17	Control	627	755
True news from a Low-Quality Source	17	Treatment	497	595
True news from a Mainstream Source	21	Control	785	1,027
True news from a Mainstream Source	21	Treatment	579	764

O Study Using Different Instructions (Study 6)

Study 6 tests if the search effects we identify on belief in false/misleading and true articles still hold when we remove the instructions we present to respondents. To this end, we ran an experiment similar to Study 1, but we add two other treatment arms in which we encourage individuals to search online to evaluate news. The three different instructions are presented below:

1. Treatment Group 1 (Full Instructions):
The purpose of this section is to find evidence from another source regarding the central claim that you're evaluating. This evidence should allow you to assess whether the central claim is true, false, or somewhere in between. Guidance for the finding evidence for or against the central claim you've identified:

(1) By evidence, we mean an article, statement, photo, video, audio, or statistic relevant to the central claim. This evidence should be reported by some other source than the author of the article you are investigating. This evidence can either support the initial claim or go against it.

(2) To find evidence about the claim, you should use a keyword search on a search engine of your choice or within the website of a particular source you trust as an authority on the topic related to the claim you're evaluating.

(3) We ask that you use the highest quality pieces of evidence to evaluate the central claim in your search. If you cannot find evidence about the claim from a source that you trust, you should try to find the most relevant evidence about the claim you can find from any source, even one you don't trust.

2. Treatment Group 2 (Full Instructions without final instruction):

The purpose of this section is to find evidence from another online source regarding the central claim that you're evaluating. This evidence should allow you to assess whether the central claim is true, false, or somewhere in between. Guidance for the finding evidence for or against the central claim you've identified:

(1) By evidence, we mean an article, statement, photo, video, audio, or statistic relevant to the central claim. This evidence should be reported by some other source than the author of the article you are investigating. This evidence can either support the initial claim or go against it.

(2) To find evidence about the claim, you should use a keyword search on a search engine of your choice or within the website of a particular source you trust as an authority on the topic related to the claim you're evaluating.

3. Treatment Group 3 (Minimal instructions):

The purpose of this section is to find evidence from another online source regarding the central claim. This evidence should allow you to assess whether the central claim is true, false, or somewhere in between.

To measure the effect of different instructions, we recruited 6,443 respondents living in the United States through Qualtrics, an online survey firm, over 10 days (August 10, 2022 to September 11, 2022) and presented them with three articles from mainstream and low-quality sources within 48 hours of publication. Participants were randomly assigned to one of the three treatment groups that encouraged the respondents to search online to help them evaluate all of the articles they were sent or were not prompted to search online.

Across forty false/misleading and true news articles, we collected 4,024 evaluations from 1,678 unique respondents in the control group, 3,846 evaluations from 1,597 unique respondents in the first treatment group, 3,833 evaluations from 1,597 unique respondents in the second treatment group, and 3,770 evaluations from 1,571 unique respondents in the third treatment group. We detail the number of evaluations for each article subset in Table S81. Details about the articles used can be found later in this section of the Supplementary Materials.

Table S81: Number of Unique Respondents and Evaluations in Study 6 by Article Type and Assigned Group

Article Type	Control or Treatment Group	Number of Unique Articles Evaluated	Number of Evaluations	Number of Unique Respondents
False/Misleading news	11	Control	1,113	1,012
False/Misleading news	11	Treatment Group 1	1,075	967
False/Misleading news	11	Treatment Group 2	1,034	952
False/Misleading news	11	Treatment Group 3	1,036	941
True news from a Low-Quality Source	12	Control	1,724	1,368
True news from a Low-Quality Source	12	Treatment Group 1	1,174	1,018
True news from a Low-Quality Source	12	Treatment Group 2	1,159	1,017
True news from a Low-Quality Source	12	Treatment Group 3	1,137	1,001
True news from a Mainstream Source	17	Control	1,187	1,029
True news from a Mainstream Source	17	Treatment Group 1	1,597	1,293
True news from a Mainstream Source	17	Treatment Group 2	1,640	1,300
True news from a Mainstream Source	17	Treatment Group 3	1,597	1,262

The figure below presents the treatment effect from Study 6 on belief in false/misleading news, true news from mainstream sources, and true news from low-quality sources using the categorical scale. The figure shows that the effect of searching online increases the probability of rating a false/misleading article as true regardless of the instructions given to respondents. The search effect on belief in false/misleading articles is smaller when fewer instructions are given (treatment groups 2 and 3), but the effect is still statistically significant at the 95 percent level. Also, when comparing the treatment groups 1 and 2 or treatment groups 1 and 3, the difference in probability a respondent rates a false/misleading article as true is not statistically significant at the 95 percent level (P=0.114 and P=0.054, respectively). The search effect on belief in true articles from mainstream and low-quality sources also shrunk, but when comparing the treatment groups 1 and 2 or treatment groups 1 and 3 the difference in probability a respondent correctly rates a true article as true is not statistically significant at the 95 percent level for true article from mainstream (P=0.118 and P=0.096, respectively) or low-quality news sources (P=0.910 and P=0.158, respectively).

Figure S6: The online search effect using different online search instructions (categorical veracity measure). This figure displays the average treatment effects and 95 percent confidence intervals for linear regression models testing the effect of SOTEN using different online search instructions in Study 6. It shows that the effect of searching online increases the probability of rating a false/misleading article as true regardless of the instructions given to respondents. When comparing the control group (N = 1,113) to treatment group 1 (N = 1,075 ; the same instructions used in Studies 1-5), treatment group 2 (N = 1,034 ; limited instructions), and treatment group 3 (N = 1,036 ; no instructions), searching online increased the likelihood of rating false/misleading news as true by 0.09 (P = 0.0027), 0.05 (P = 0.0389), and 0.05 (P = 0.0021) respectively. The effects of online search were similar for true news from mainstream sources and true news from low-quality sources.

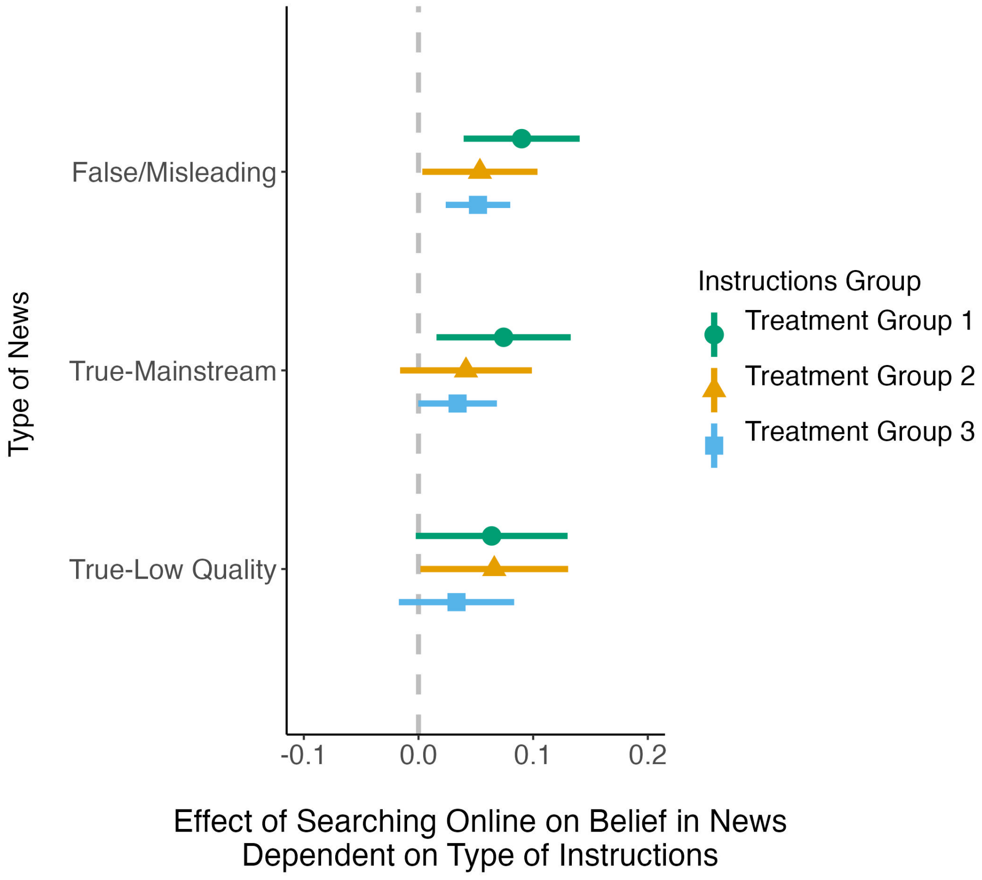

Articles Exposed to Respondents (Study 6)

Table S82: Headlines for Articles Chosen from the Low Quality Conservative News Stream in Study 6

Date	Headline	Modal Fact Checker Rating	Topic	Lean of Article
8/15/22	Happy birthday, Anne! Queen's matter of fact message to her daughter on her birthday	True	Human Interest	Unclear
8/16/22	Fed-up Ron DeSantis sends police to remove WOKE Soros-backed state prosecutor from office	Could Not Determine	Politics	Conservative
8/22/22	Report Says Who's Next On The CNN Chopping Block After Stelter Ouster	Could Not Determine	Politics	Conservative
8/23/22	Eurozone in freefall as EU currency hits new 20-year low – Euro now worth LESS than Dollar	True	Economics	Unclear
8/24/22	JUST IN: Ford Announces It Will Cut THOUSANDS of Jobs In MI...Here's Why Joe Biden and Michigan's Incompetent Gov Gretchen Whitmer Are To Blame	False/Misleading	Economics	Conservative
8/29/22	European Natural Gas Prices Are 6 Times Higher Than Last Year, And This Is Sparking Widespread Civil Unrest All Over Europe	False/Misleading	Economics	Unclear
8/30/22	It's all RIGGED beyond imagination: Deep state pushing for "blood in the streets" across America before Halloween – DON'T FALL FOR IT	False/Misleading	Politics	Conservative
8/31/22	'Terrible President. Even Worse Human Being': Lisa Boothe Tears Into Biden	Could Not Determine	Politics	Conservative
9/5/22	You Can't Make This Up: Terrence Williams Put His Own Picture on the Box of his Pancake Mix — Facebook Labeled it as 'Racist'	Could Not Determine	Human Interest	Conservative
9/6/22	More Evidence Revealed of Biden's Involvement in FBI Action Against Trump	False/Misleading	Politics	Conservative

Table S83: Headlines for Articles Chosen from the Low Quality Liberal News Stream in Study 6

Date	Headline	Modal Fact Checker Rating	Topic	Lean of Article
8/15/22	Rand Paul Announces We Should Repeal The Espionage Act	True	Politics	Liberal
8/16/22	Donald Trump says the DOJ has seized his passports	False/Misleading	Politics	Liberal
8/22/22	Liz Cheney Publicly Shames Ted Cruz For Betraying America	True	Politics	Liberal
8/23/22	Another Defamation Lawsuit Against Alex Jones Ordred To Proceed	True	Politics	Unclear
8/24/22	ULTIMATE HYPOCRISY: Florida Sen. Rick Scott slams Biden's Delaware vacation from a yacht in Italy	False/Misleading	Politics	Liberal
8/29/22	Postal Employees Speak Out Against Louis DeJoy For Weakening USPS	Could Not Determine	Politics	Unclear
8/30/22	Conservatives Have A Fit Over Armed Citizens Guarding Drag Event	True	Politics	Liberal
8/31/22	GAME OVER	False/Misleading	Politics	Liberal
9/5/22	Ukraine Counter-Strike Wipes Out Almost 500 Russian Troops In A Day	Could Not Determine	Politics	Liberal
9/6/22	DEFENDING DEMOCRACY: Biden continues attack on MAGA Republicans in Wisconsin speech	True	Politics	Liberal

Table S84: Headlines for Articles Chosen from the Low Quality Unclear Partisanship News Stream in Study 6

Date	Headline	Modal Fact Checker Rating	Topic	Lean of Article
8/15/22	ALARMING new evidence suggests monkeypox is cover-up for COVID jab-induced adverse effect	False/Misleading	Science	Unclear
8/16/22	DeSantis Takes Stage, Promises What's Happening 'Will Result in the Retirement of Nancy Pelosi'	True	Politics	Conservative
8/22/22	Dennis Rodman Says He's Been Given Permission to Travel to Russia to Seek Release of Brittney Griner	True	Human Interest	Neutral
8/23/22	World Economic Forum Suggests There Are "Solid, Rational" Reasons To Microchip Kids	False/Misleading	Science	Unclear
8/24/22	"Tsunami Of Shutoffs": 1 In 6 US Homes Are Behind On Power Bills	True	Economics	Conservative
8/29/22	'Bring Me To Life' By Evanescence Just Reached Number One On iTunes in the US Out of Nowhere	True	Human interest	Neutral
8/30/22	News Alert: FBI Agent Out After Accusations of Political Bias Suppressing Hunter Biden Laptop	False/Misleading	Politics	Conservative
8/31/22	"How In The Name Of God": Shocked Europeans Post Astronomical Energy Bills As 'Terrifying Winter' Approaches	True	Economics	Unclear
9/5/22	Fearing a Trump Smack-Down, Nation's Top Networks Refused to Air Biden Speech	False/Misleading	Politics	Conservative
9/6/22	Surprise New Poll Shows State That Hasn't Had a Republican Senator in Over 20 Years Could Flip Red	Could Not Determine	Politics	Conservative

Table S85: Headlines for Articles Chosen from the Conservative Mainstream News Stream in Study 6

Date	Headline	Modal Fact Checker Rating	Topic	Lean of Article
8/15/22	Leonardo DiCaprio funneled grants through dark money group to fund climate nuisance lawsuits	Could Not Determine	No Mode	Conservative
8/16/22	Trump's call for transparency, John Kerry's far-left 'climate aristocracy' and more top headlines	Could Not Determine	Politics	Conservative
8/22/22	'We had a lightning strike': 911 calls released after Florida mother, child hit	True	Human Interest	Neutral
8/23/22	Dozens of dogs killed by mysterious parvo-like illness in Michigan	True	No Mode	Neutral
8/24/22	Trump celebrates 'ALL' endorsement wins in primary: 'Great candidates!'	True	Politics	Unclear
8/29/22	New York stores begin enforcing ban on sale of whipped cream canisters to those under 21	True	Human Interest	Neutral
8/30/22	Border Patrol chief says border crisis a result of Biden's 'no consequences' policy for illegal migrants	Could Not Determine	Politics	Conservative
8/31/22	6-year-old Alexa changes name due to Amazon-related bullying	True	Human Interest	Neutral
9/5/22	Mayor Lori Lightfoot lashes out at Texas Gov. Abbott after 50 more migrants are bussed to Chicago	True	Politics	Conservative
9/6/22	Cops confirm body found in Memphis is kidnapped heiress Eliza Fletcher	True	Neutral	Neutral/Unclear

Table S86: Headlines for Articles Chosen from the Liberal Mainstream News Stream in Study 6

Date	Headline	Modal Fact Checker Rating	Topic	Lean of Article
8/15/22	Covid vaccine designed to target two variants approved for use in UK	True	Science	Neutral
8/16/22	Trump's initially 'upbeat' mood about the FBI's Mar-a-Lago raid turned dark when GOP support began to wane, report says	True	Politics	Neutral
8/22/22	Apple workers launch petition over company's reported return-to-office plan	True	Economics	Neutral
8/23/22	A drone that flew for over two months in a US Army test crashed in Arizona after nearly breaking a record for the longest flight	True	Science	Neutral
8/24/22	Democrat Pat Ryan wins bellwether special election in New York's Hudson Valley	True	Politics	Neutral
8/29/22	Sen. Lindsey Graham said if Trump is prosecuted for mishandling classified information 'there will be riots in the streets'	True	Politics	Neutral
8/30/22	Alan Dershowitz says lawyers are telling him they won't defend Trump because they don't want to be ostracized or 'canceled'	True	Politics	Unclear
8/31/22	Crist to resign from Congress as race for Florida governor heats up	True	Politics	Neutral
9/5/22	GOP Consultant Says Biden's Anti-MAGA Speech Made Her Cry, Gave Her Hope	True	Politics	Liberal
9/6/22	An influencer and OnlyFans star is facing backlash after saying 'nobody wants to work these days'	True	Unclear	Neutral/Unclear

Balance Tables (Study 6)

Table S87: Balance Table for Those in the Control and Treatment Group 1 in Study 6. To determine whether the difference between each group was statistically significant we fit an OLS regression model to predict each demographic variable using a dummy variable denoting whether the respondent was in the treatment group or not.

Demographic	Average (Treatment)	Average (Control)	Difference
Education	2.16	2.02	0.14***
Age	50.96	55.08	-4.12***
Gender	0.34	0.35	-0.01
Income	0.79	0.75	0.04
Ideology	-0.09	0.01	-0.1

$^{***}p < 0.001$, $^{**}p < 0.01$, $^{*}p < 0.05$

Table S88: Balance Table for Those in the Control and Treatment Group 2 in Study 6. To determine whether the difference between each group was statistically significant we fit an OLS regression model to predict each demographic variable using a dummy variable denoting whether the respondent was in the treatment group or not.

Demographic	Average (Treatment)	Average (Control)	Difference
Education	2.15	2.02	0.13***
Age	50.9	55.08	-4.18***
Gender	0.35	0.35	0
Income	0.76	0.75	0.01
Ideology	-0.03	0.01	-0.04

$^{***}p < 0.001$, $^{**}p < 0.01$, $^{*}p < 0.05$

Table S89: Balance Table for Those in the Control and Treatment Group 3 in Study 6. To determine whether the difference between each group was statistically significant we fit an OLS regression model to predict each demographic variable using a dummy variable denoting whether the respondent was in the treatment group or not.

Demographic	Average (Treatment)	Average (Control)	Difference
Education	2.06	2.02	0.04
Age	49.89	55.08	-5.19***
Gender	0.36	0.35	0.01
Income	0.77	0.75	0.02
Ideology	-0.04	0.01	-0.05

$^{***}p < 0.001$, $^{**}p < 0.01$, $^{*}p < 0.05$

P Search Terms and Quality of News Sources Returned

Article: “U.S. faces engineered famine as COVID lockdowns and vax mandates could lead to widespread hunger, unrest this winter,” the term “engineered famine”

Table S90: Search Terms and Quality of Information Returned

Search Term	Exposed to Unreliable Result	Ideologically Congruent	Digital Literacy Score
biden infrastructure food supply chain issues	0	0	53
%22engineered famine%22 COVID	1	1	59
biden infrastructure supply chain issues	0	0	53
covid 19 panic buying	0	1	66
Rep. Sam Graves supply chain issues	0	0	53
Biden regime leads to food shortage	0	0	49
us food shortage 2021	0	0	62
supply chain mess	0	0	56
https%3A%2F%2Fwww.naturalnews.com%2F2021-11-01-us-faces-engineered-famine-covid-lockdowns-vax-mandates.html	1	1	51
covid supply chain explaination	0	0	57
U.S. faces engineered famine as COVID lockdowns and vax mandates could lead to widespread hunger%2C unrest this winter	1	1	51
united states food shortage 2021	0	0	52
democrats causing supply chain crisis	0	1	51
us faces engineered famine covid	1	0	58
engineered famine 2021	0	0	52
Engineered Famine	0	0	66
engineered famine 2021	0	1	51
%22engineered famine%22 COVID hoarding	1	1	59
Are the food supply issues manufactured	0	0	64
grocery stores restocking	0	1	53
biden infrastructure food supply chain issues	0	0	53
SHIPPING BOTTLENECKS 2022	0	0	52
grocery store market empty famine	0	1	53
food shortages this winter supply chain	0	0	55
engineered famine	0	1	53
us food shortage 2021	0	0	62
food crisis in US winter 2021	0	0	54
U.S. faces engineered famine as COVID lockdowns and vax mandates could lead to widespread hunger%2C unrest this winter	1	0	46
US famine winter 2021	0	0	54
is the covid famine on purpose	0	0	57
usa famine because of covid lockdown	0	0	34
famine covid lockdown	0	0	34
Covid famine	0	0	51
supply chain issues	0	0	56

Table S91: Search Terms and Quality of Information Returned

Search Term	Exposed to Unreliable Result	Ideologically Congruent	Digital Literacy Score
united states food shortage 2021	0	0	52
engineered famine COVID	1	1	53
U.S. faces engineered famine as COVID lockdowns and vax mandates could lead to widespread hunger%2C unrest this winter	1	0	57
engineered famine 2021 in America	0	1	51
explain covid famine	0	0	57
engineered famine	0	1	59
engineered famine hunger	0	0	54
US faces engineered famine	1	1	54
covid widespread hunger shortages	0	1	31
biden infrastructure supply chain shortage fact check	0	0	53
supply chain holidays	0	0	56
u.s. supply chain problems	0	0	56
food shortage	1	0	42
us food supply shortage statistics	0	1	48
us food supply shortage	0	1	48
U.S. COVID lockdowns and vax mandates could lead to widespread hunger	0	0	54
U.S. faces engineered famine as COVID lockdowns and vax mandates could lead to widespread hunger%2C unrest this winter	1	1	53
U.S. faces engineered famine COVID	1	1	53
Biden infrastructure supply chain issues	0	0	53
engineered famine in the united states 2021	0	0	58
is joe biden behind the slow down in supply chain	0	1	61
U.S. faces famine	0	0	53
natural news engineered famine	1	1	53
us food shortage 2021	0	0	62
us food shortage 2021	0	0	62
U.S. engineered famine	0	0	61
U.S. faces engineered famine as COVID lockdowns	1	1	35
us food shortage 2021	0	0	62
us food shortage 2021	0	0	62
us food shortage 2021	0	0	62
us food shortage 2021	0	0	62
covid engineered famine	0	0	57
U.S. faces engineered famine	0	1	65
U.S. faces engineered famine as COVID lockdowns and vax mandates could lead to widespread hunger%2C unrest this winter	1	0	57
engineered famine as COVID lockdowns and vax mandates could lead to widespread hunger%2C	1	0	58
food shortage	1	0	61
U.S. faces engineered famine as COVID lockdowns and vax mandates could lead to widespread hunger%2C unrest this winter	1	0	54
%22engineered famine%22	0	1	59
are supermarkets empty	0	1	53
will the us face famine	0	1	53
us food supply	0	1	48

Table S92: Search Terms and Quality of Information Returned

Search Term	Exposed to Unreliable Result	Ideologically Congruent	Digital Literacy Score
Covid19 causing famine	0	1	54
grocery shelves empty again	0	1	53
us food shortage 2021	0	0	62
food shortages supply chain	0	1	63
what is causing high food prices	0	1	63
grocery shelves empty again	0	1	53
COVID lockdowns and vax mandates could lead to widespread hunger	1	0	60
world hunger statistics 2020	0	1	65
U.S. faces engineered famine as COVID lockdowns and vax mandates could lead to widespread hunger%2C unrest this winter	1	0	43
us food shortage 2021	0	0	62
food shortages in america 2021	0	0	41
us food shortage 2021	0	0	41
food shortage this winter	0	0	58
U.S. faces engineered famine as COVID lockdowns and vax mandates could lead to widespread hunger%2C unrest this winter	1	0	57
covid engineered famine	0	0	57
us food shortage 2021	0	0	62
biden infrastructure supply chain issues	0	0	53
Engineered Famine Covid	1	0	66
biden infrastructure food supply chain issues	0	0	53
U.S. faces engineered famine as COVID lockdowns and vax mandates could lead to widespread hunger%2C unrest this winter	1	1	38
U.S. faces engineered famine as COVID lockdowns and vax mandates could lead to widespread hunger%2C unrest this winter	1	0	45
covid food shortages	0	1	31
covid 19 panic buying	0	1	66
biden infrastructure food supply chain issues fact check	0	0	53
covid lockdowns USA engineered famine	1	0	51
U.S. faces famine	0	0	53
U.S. engineered famine	0	0	61
U.S. faces famine in 2021	0	0	53
is the us facing engineered famine	0	1	65
hunger and food shortage in the US	0	0	54
covid famine in the USA	0	0	57
COVID famines USA	0	0	60
is there a supply chain crisis affecting food supply	0	1	61
food shortage usa	0	0	42
biden infrastructure supply chain issues	0	0	53
food shortage winter 2022	0	0	52
biden infrastructure supply chain issues fact check	0	0	53
pandemic supply chain	0	0	57
2021 famine prediction	0	1	65
U.S. faces engineered famine	1	1	65
U.S. faces engineered famine	1	1	65
covid 19 panic buying	1	1	66
famine due to covid lockdowns and vaccine mandates	0	0	62
us Engineered Famine	0	1	54
america faces famine in 2021	0	0	53
us food shortage 2021	0	0	62
covid lockdowns USA engineered famine	1	0	51

Article: “Looks like New York prosecutors have a witness directly incriminating Donald Trump.”

Table S93: Search Terms and Quality of Information Returned

Search Term	Exposed to Unreliable Result	Ideologically Congruent	Digital Literacy Score
jennifer Weisselberg trump incriminate	0	0	49
jennifer weisselberg incrimination trump	0	0	55
weisselberg trump indictment	0	1	66
New York prosecutors have a witness directly incriminating Donald Trump	1	0	48
Jennifer Weisselberg and Trump fake news	0	1	57
weisselberg daughter in law testimony	0	1	61
Looks like New York prosecutors have a witness directly incriminating Donald Trump	1	1	64
Palmer REport indicting trump	1	1	65
witness trump	0	1	62
Looks like New York prosecutors have a witness directly incriminating Donald Trump	1	0	46
Jennifer Weisselberg and Trump	0	1	57
Jennifer Weisselberg%E2%80%99s testimony	0	1	60
New York prosecutors have a witness directly incriminating Donald Trump	1	0	66
weisselberg daughter-in-law	0	1	61
New York prosecutors have a witness directly incriminating Donald Trump	1	1	45
NY indicting trump	0	1	65
Allen Weisselberg trump incriminate	0	0	49
Allen Weisselberg trump incriminate	0	0	49
New York Prosecuters inditing donald trump	0	0	57
Jennifer Weisselberg and Trump	0	1	57
Jennifer Weisselberg and Trump	0	1	57
New York prosecutors have a witness directly incriminating Donald Trump	1	0	32

Table S94: Search Terms and Quality of Information Returned

Search Term	Exposed to Unreliable Result	Ideologically Congruent	Digital Literacy Score
Jennifer Weisselberg and Trump fox news	0	1	57
Weisselberg trump news jennifer	0	1	45
Jennifer Weisselberg	0	1	58
Weisselberg trump news	0	1	45
prosecutors have a witness directly incriminating Donald Trump	1	1	56
Will Jennifer Weisslberg testify against trump	0	0	58
what has jennifer weisselberg testified	0	0	64
Jennifer Weisselberg and Trump fake news	0	1	57
Jennifer Weisselberg witness Trump trial	0	0	62
Jennifer Weisselberg trump allegations	0	0	59
Looks like New York prosecutors have a witness directly incriminating Donald Trump	1	1	45
NY indicting trump	0	1	65
jennifer weisselberg	0	0	61
Jennifer Weisselberg testimony NY prosecutors Trump	0	0	60

Q Balance Tables

Table S95: Balance Table for those in the Control and Treatment Group in Study 1. To determine whether the difference between each group was statistically significant we fit an OLS regression model to predict each demographic variable using a dummy variable denoting whether the respondent was in the treatment group or not.

Demographic	Average (Treatment)	Average (Control)	Difference
Education	2.32	2.38	-0.06
Age	45.12	46.26	-1.14
Gender	0.47	0.5	-0.03
Income	0.78	1.1	-0.32***
Ideology	-0.08	0	-0.08

$^{***}p < 0.001$, $^{**}p < 0.01$, $^{*}p < 0.05$

Table S96: Summary Statistic of participants in Study 2

Demographic	Mean	Standard Deviation
Education	2.31	1.23
Age	45.44	16.2
Gender (Female)	0.5	0.5
Income	0.95	0.98
Ideology	0	1.72

Table S97: Summary Statistic of participants in Study 3

Demographic	Mean	Standard Deviation
Education	2.37	1.2
Age	44.04	16.36
Gender (Female)	0.49	0.5
Income	0.95	0.95
Ideology	-0.04	1.76

Table S98: Summary Statistic of participants in Study 4

Demographic	Mean	Standard Deviation
Education	2.34	1.24
Age	44.57	17.43
Gender (Female)	0.47	0.5
Income	1.03	0.99
Ideology	0.01	1.74

Table S99: Balance Table for Those in the Control and Treatment Group in Study 5. To determine whether the difference between each group was statistically significant we fit an OLS regression model to predict each demographic variable using a dummy variable denoting whether the respondent was in the treatment group or not.

Demographic	Average (Treatment)	Average (Control)	Difference
Education	3.48	3.5	-0.02
Age	37.07	39.24	-2.17**
Gender (Female)	0.43	0.47	-0.04
Income	1.74	1.79	-0.05
Ideology	-0.38	-0.5	0.12

$^{***}p < 0.001$, $^{**}p < 0.01$, $^{*}p < 0.05$

Table S100: Balance Table: Compliers and Non-Compliers in Treatment Group in Study 5. To determine whether the difference between each group was statistically significant we fit an OLS regression model to predict each demographic variable using a dummy variable denoting whether the respondent complied or not.

Demographic	Average (Complier)	Average (Non-Complier)	Difference
Education	3.42	3.67	-0.25**
Age	36.6	38.86	-2.26*
Gender	0.42	0.5	-0.08
Income	1.73	1.74	-0.01
Ideology	-0.61	0.19	-0.8***
Digital Literacy	55.14	49.28	5.86***

$^{***}p < 0.001$, $^{**}p < 0.01$, $^{*}p < 0.05$

Table S101: Balance Table: Compliers and Non-Compliers in Control Group. To determine whether the difference between each group was statistically significant we fit an OLS regression model to predict each demographic variable using a dummy variable denoting whether the respondent complied or not.

Demographic	Average (Complier)	Average (Non-Complier)	Difference
Education	3.49	3.87	-0.38
Age	39.13	42.76	-3.63
Gender (Female)	0.47	0.52	-0.05
Income	1.8	1.81	-0.01
Ideology	-0.57	-0.02	-0.55*
Digital Literacy	54.81	48.59	6.22***

$^{***}p < 0.001$, $^{**}p < 0.01$, $^{*}p < 0.05$

Table S102: All Respondents with Digital Trace Data in Control and Treatment Group. To determine whether the difference between each group was statistically significant we fit an OLS regression model to predict each demographic variable using a dummy variable denoting whether the respondent was in the treatment group or not.

Demographic	Average (Treatment)	Average (Control)	Difference
Education	3.42	3.49	-0.07
Age	36.6	39.13	-2.53***
Gender	0.42	0.47	-0.05*
Income	1.73	1.8	-0.07
Ideology	-0.61	-0.57	-0.04
Digital Literacy	55.14	54.81	0.33

$^{***}p < 0.001$, $^{**}p < 0.01$, $^{*}p < 0.05$

References

Guess, A., Nyhan, B., & Reifler, J. (2020). Exposure to untrustworthy websites in the 2016 us election. *Nature Human Behaviour*, *4*(5), 472–480.

MacInnis, B., Krosnick, J. A., Ho, A. S., & Cho, M.-J. (2018). The accuracy of measurements with probability and nonprobability survey samples: Replication and extension. *Public Opinion Quarterly*, *82*(4), 707–744.

Mullinix, K. J., Leeper, T. J., Druckman, J. N., & Freese, J. (2015). The generalizability of survey experiments. *Journal of Experimental Political Science*, *2*(2), 109–138.

Zack, E. S., Kennedy, J., & Long, J. S. (2019). Can nonprobability samples be used for social science research? a cautionary tale. *Survey Research Methods*, *13*(2), 215–227.
