# Supplementary material for: Online searches to evaluate misinformation can increase its perceived veracity
Source: Nature. 2023 Dec 20;625(7995):548–56. doi: 10.1038/s41586-023-06883-y (PMC10794132; doi:10.1038/s41586-023-06883-y)
Supplement: Supplementary file 2 — Reporting Summary [file 41586_2023_6883_MOESM2_ESM.pdf]

nature portfolio

Corresponding author(s): Kevin Aslett

Last updated by author(s): Oct 10, 2023

Reporting Summary

Nature Portfolio wishes to improve the reproducibility of the work that we publish. This form provides structure for consistency and transparency in reporting. For further information on Nature Portfolio policies, see our Editorial Policies and the Editorial Policy Checklist.

Statistics

For all statistical analyses, confirm that the following items are present in the figure legend, table legend, main text, or Methods section.

n/a	Confirmed	
☐	☒	The exact sample size (*n*) for each experimental group/condition, given as a discrete number and unit of measurement
☐	☒	A statement on whether measurements were taken from distinct samples or whether the same sample was measured repeatedly
☐	☒	The statistical test(s) used AND whether they are one- or two-sided<br>*Only common tests should be described solely by name; describe more complex techniques in the Methods section.*
☐	☒	A description of all covariates tested
☐	☒	A description of any assumptions or corrections, such as tests of normality and adjustment for multiple comparisons
☐	☒	A full description of the statistical parameters including central tendency (e.g. means) or other basic estimates (e.g. regression coefficient) AND variation (e.g. standard deviation) or associated estimates of uncertainty (e.g. confidence intervals)
☐	☒	For null hypothesis testing, the test statistic (e.g. *F*, *t*, *r*) with confidence intervals, effect sizes, degrees of freedom and *P* value noted<br>*Give P values as exact values whenever suitable.*
☒	☐	For Bayesian analysis, information on the choice of priors and Markov chain Monte Carlo settings
☐	☒	For hierarchical and complex designs, identification of the appropriate level for tests and full reporting of outcomes
☐	☒	Estimates of effect sizes (e.g. Cohen's *d*, Pearson's *r*), indicating how they were calculated

Our web collection on statistics for biologists contains articles on many of the points above.

Software and code

Policy information about availability of computer code

Data collection	Two web extensions were used in the data collection process for Study 5. They are titled: "Search Engine Results Saver" and the "URL Historian." They are available on the chrome webstore for free. No other code or software was used.
Data analysis	R (4.2.3) and RStudio (2023.03.0+386) was used to clean and analyze the data. We created our own code to do so.

For manuscripts utilizing custom algorithms or software that are central to the research but not yet described in published literature, software must be made available to editors and reviewers. We strongly encourage code deposition in a community repository (e.g. GitHub). See the Nature Portfolio guidelines for submitting code & software for further information.

Data

Policy information about availability of data

All manuscripts must include a data availability statement. This statement should provide the following information, where applicable:

- Accession codes, unique identifiers, or web links for publicly available datasets
- A description of any restrictions on data availability
- For clinical datasets or third party data, please ensure that the statement adheres to our policy

Data and materials for all of the studies are available at https://github.com/SMAPPNYU/Do_Your_Own_Research.

Research involving human participants, their data, or biological material

Policy information about studies with human participants or human data. See also policy information about sex, gender (identity/presentation), and sexual orientation and race, ethnicity and racism.

Reporting on sex and gender	Gender was used as a control variable in some of the analyses conducted in the paper. Gender was determined by self-reporting. We do provide disaggregated gender data in the source data. We do not find any evidence in our studies that the findings only applied to one sex or gender. We did not pre-register any hypotheses regarding gender and we stuck close to our pre-registration.
Reporting on race, ethnicity, or other socially relevant groupings	Participants themselves provided demographic information that we controlled for in our study. We controlled for the following variables: age, education, income, political ideology, and gender. The following questions asked individuals for this data:<br><br>Age: What is your age?<br><br>Ecucation: We asked individuals to self-identify their highest degree earned.<br><br>Income: We asked individuals to self-identify their income from last year.<br><br>Political Ideology: Where would you place yourself on this scale? Extremely Conservative - Extremely Liberal<br><br>Gender: What is your gender?<br><br>We ran experimental studies that sampled a representative sample of individuals using quota-sampling and randomized the treatment. We then controlled for these demographic variables to improve the precision of our average treatment effect.
Population characteristics	In all of the studies we sampled individuals living in the United States. In the first four of our studies and the sixth study these individuals were recruited by Qualtrics. These samples were representative. We quote-sample respondents based on age, gender, and education. The sample for the fifth study was recruited using Mechanical Turk. This sample was not representative and was not quota-sample based on demographic variables. Balance tables for each study including this demographic information is listed in the methods section of the main text.<br><br>By sampling individuals through online opt-in surveys we do understand that we are oversampling highly online individuals, but this is our target population. We are most interested in frequent users of the internet who are most likely to consume online news.
Recruitment	Participants were recruited by Qualtrics, an online survey platform, and Amazon's Mechanical Turk. In both of these cases, participants were told what they would be asked to do in the survey and could opt out at any time. Given these internet surveys use opt-in panels and we know they are the less accurate than probability sampling, we must be cautious when reporting our results. For example, we expect the behavior of our respondents who self-selected into the survey to differ from those drawn with known probability from a well-specified population. Therefore, it is possible and likely this convenience sample is different in possibly unmeasured ways. Therefore, we only report results from analyses with that we can using non-probability sampling.<br><br>Although we should be cautious when making experimental inferences using an opt-in non-probability samples from Qualtrics, previous work has found that about 90% of effects identified using a gold-standard probability sample are similar to effects identified by an opt-in Qualtrics panel.<br><br>A major issue in online opt-in surveys is that the behaviors of those who opt-in to and join multiple panels to earn incentives may put much less effort into tasks at hand and are more likely to guess to save time and maximize their payment. To test if this would affect our main results we ran a parallel survey and paid respondents additional payments for correct answers to our veracity question, but did not find much of any difference in their responses. Therefore, we do not believe that a lack of effort explains the results we find. Recent work has also shown that experimental results from these non-probability samples are often comparable to those found in population samples. Given this previous work, the results we present are not likely to be different if we had used probability-sampling.<br><br>An additional possible issue is that we may have different levels of attrition in the control and treatment groups in a few of our studies. We report dropout levels and balance tables for every study in our paper to provide evidence that we do not believe this to be an issue.<br><br>An added advantage of using online sampling is that it predominately recruits those in whom we are actually most interested: in, frequent users of the internet who are most likely to consume online news. Thus even if our results are less likely to be generalizable to overall population, they are still likely to be generalizable to the population that consumes news online more rather than other recruiting techniques such as in-person surveys.
Ethics oversight	Study 1 was approved by NYU IRB protocol IRB-FY2019-3511<br>Study 2 was approved by NYU IRB protocol IRB-FY2019-3511<br>Study 3 was approved by NYU IRB protocol IRB-FY2019-3511<br>Study 4 was approved by NYU IRB protocol IRB-FY2019-3511<br>Study 5 was approved by NYU IRB protocol IRB-FY2021-5608<br>Study 6 was approved by a modified NYU IRB protocol IRB-FY2019-3511<br>We received informed consent from all participants in Studies 1-6.

Note that full information on the approval of the study protocol must also be provided in the manuscript.

Field-specific reporting

Please select the one below that is the best fit for your research. If you are not sure, read the appropriate sections before making your selection.

☐ Life sciences ☒ Behavioural & social sciences ☐ Ecological, evolutionary & environmental sciences

For a reference copy of the document with all sections, see nature.com/documents/nr-reporting-summary-flat.pdf

Behavioural & social sciences study design

All studies must disclose on these points even when the disclosure is negative.

Study description

Quantitative Experimental Studies.

In Study 1, we tested whether SOTEN affects belief in misinformation in a randomized controlled trial that ran for ten days. During this study, we asked two different groups of respondents to evaluate the same false/misleading or true articles in the same 24-hour window, but only one after searching online.

Study 2 ran similarly to Study 1, but over 29 days between November 18, 2019 and February 6, 2020. In each survey that was sent in Study 1, we asked respondents in the control group to evaluate the third article they received a second time, but only after looking for evidence online (using the same directions to search online that participants in Study 1 received).

Study 3 replicated Study 2 using the same materials and procedure, but was run between March 16, 2020 and April 28, 2020, three to five months after the publication of each these articles.

Study 4 extended Study 2 by asking individuals to evaluate and re-evaluate highly popular misinformation strictly about Covid-19 after searching online. This study was run over eight days between May 28, 2020 to June 22, 2020.

Study 5 was almost identical to Study 1, but we used a custom plug-in to collect digital trace data and encouraged respondents to specifically search online using Google (our web browser plug-in could only collect search results from a Google search result page). Similar to Study 1, we measured the effect of SOTEN on belief in misinformation in a randomized controlled trial that ran on twelve separate days from July 13, 2021 to November 9, 2021, during which we asked two different groups of respondents to evaluate the same false/misleading or true articles in the same 24-hour window. The treatment group was encouraged to search online, while the control group was not.

Study 6 tests if the search effects we identify on belief in false/misleading and true articles still hold when we remove the instructions we present to respondents. To this end, we ran an experiment similar to Study 1, but we add two other treatment arms in which we encourage individuals to search online to evaluate news.

Research sample

In the first four of our studies our sample of those living in the United States is recruited by Qualtrics. The sample is representative. We quote-sample respondents based on age, gender, and education. The final study was recruited using Mechanical Turk. This sample was not representative and was not quota-sample based on demographic variables.

By sampling individuals through online opt-in surveys we do understand that we are oversampling highly online individuals, but this is our target population. We are most interested in, frequent users of the internet who are most likely to consume online news.

Balance tables for each study including this demographic information is listed in the methods section of the main text.

Sampling strategy

Individuals are randomly sampled. No statistical methods were used to predetermine sample size. Generally the sample sizes used (N > 1000) are large enough to identify small effects (Cohen's D above 0.2) using our models.

Data collection

Respondents took these surveys online from either their desktop or mobile phone. The respondents did not interact with the researcher.

Timing

The timing of each study can be found below:
Study 1: November 21st, 2019 to January 7, 2020
Study 2: November 18th, 2019 to February 6th, 2020
Study 3: May 28th, 2020 to June 22nd, 2020
Study 4: March 16th, 2020 to April 28th, 2020
Study 5: July 13th, 2021 to November 9th, 2021
Study 6: August 10th, 2022 to September 11th, 2022

Data exclusions

No data was excluded from the analysis.

Non-participation

We report varied levels of non-participation in our five studies. These non-participants could have declined to participate in the survey, dropped out after starting the survey, or were dropped because they failed an attention check. Participants who declined to participate or dropped out of the study, did not notify us why they refused to participate. The percentage of non-participation can be found below:
Study 1: 82%
Study 2: 82%

	Study 3: 74%<br>Study 4: 76%<br>Study 5: 75%<br>Study 6 (only reported in supplementary materials): 78%
Randomization	Individuals were randomly allocated to different experimental groups.

Reporting for specific materials, systems and methods

We require information from authors about some types of materials, experimental systems and methods used in many studies. Here, indicate whether each material, system or method listed is relevant to your study. If you are not sure if a list item applies to your research, read the appropriate section before selecting a response.

Materials & experimental systems

n/a	Involved in the study
☒	☐ Antibodies
☒	☐ Eukaryotic cell lines
☒	☐ Palaeontology and archaeology
☒	☐ Animals and other organisms
☒	☐ Clinical data
☒	☐ Dual use research of concern
☒	☐ Plants

Methods

n/a	Involved in the study
☒	☐ ChIP-seq
☒	☐ Flow cytometry
☒	☐ MRI-based neuroimaging
